# Targeting oncogenic activation of FLT3/SREBP/FASN promotes the therapeutic effect of quizartinib involving disruption of mitochondrial phospholipids

**DOI:** 10.1038/s41419-025-07661-6

**Published:** 2025-04-22

**Authors:** Feng Yin, Jing Yang, Hao Luo, Tiantian Yu, Wenhua Lu, Mingyue Zhao, Hongli Du, Shijun Wen, Peng Huang, Yumin Hu

**Affiliations:** 1 State Key Laboratory of Oncology in South China, Guangdong Provincial Clinical Research Center for Cancer, Cancer Metabolism and Intervention Research Center, Sun Yat-sen University Cancer Center, Guangzhou, 510060 Guangdong China; 2 Metabolomics Research Center, Sun Yat-sen University Zhongshan School of Medicine, Guangzhou, 510080 China; 3 School of Biology and Biological Engineering, South China University of Technology, Guangzhou, 510006 Guangdong China

**Keywords:** Cancer metabolism, Targeted therapies

## Abstract

FMS-like tyrosine kinase 3–internal tandem duplication (FLT3/ITD) is a common driver mutation that presents with a high leukemic burden and its impact on metabolic homeostasis remains to be further investigated. Here, we revealed that the oncogenic activation of FLT3/ITD induced upregulation of target genes of sterol regulatory element-binding proteins (SREBPs) in vivo and in acute myeloid leukemia patients. Quizartinib is a second-generation FLT3 inhibitor that selectively inhibits the activating FLT3 mutations. We demonstrated the critical role of SREBP1 degradation in conferring the response of FLT3/ITD cells to quizartinib. Mechanistically, quizartinib facilitated degradation of the precursor form of SREBP1 via the FLT3/AKT/GSK3 axis and reduced protein levels of its target gene fatty acid synthase (FASN). Lipidomics analysis by Liquid Chromatography Mass Spectrometry (LC-MS) demonstrated that inhibition of FLT3 altered global levels of phospholipids including reduction of cardiolipin, leading to subsequent loss of mitochondrial membrane potential. Pharmacological inhibition of SREBP1 or FASN sensitized FLT3/ITD leukemia cells to quizartinib. Quizartinib combined with SREBP inhibitor fatostatin or FASN inhibitor orlistat provided substantial therapeutic benefit over monotherapies in the murine FLT3/ITD leukemia model. Our results indicated the mechanistic link between FLT3/ITD and SREBP degradation and suggested the combination therapy via targeting FLT3/SREBP/FASN axis.

## Introduction

Internal tandem duplication in FMS-like tyrosine kinase 3 (FLT3/ITD) is a common type of activating mutation and frequently detected in acute myeloid leukemia (AML) [1]. We previously demonstrated that FLT3/ITD caused activation of glycolytic activity [2]. Others have shown glutamine metabolism associated with resistance to FLT3-targeted therapy [3, 4]. FLT3/ITD–driven leukemia has also been shown to be dependent upon serine for proliferation and survival [5]. The functional importance of lipid metabolism in FLT3/ITD leukemia has remained to be further investigated. Identification of additional FLT3/ITD-specific metabolic vulnerabilities may offer new therapeutic opportunities for this refractory subtype of leukemia.

Sterol regulatory element-binding proteins (SREBPs) are a family of transcription factors known as master regulators of cholesterol and lipid metabolism. While the isoforms of SREBP-1 mainly promote gene transcription of fatty acid synthesis [6], SREBP-2 mainly regulates cholesterol synthesis [7]. SREBPs initially exist as membrane-bound precursors within the endoplasmic reticulum (ER). Their activation involves transportation to the Golgi apparatus facilitated by the escort protein known as SREBP cleavage-activating protein (SCAP). Upon reaching the Golgi apparatus, SREBPs undergo proteolytic cleavage, releasing their active NH2 terminal domain [8]. This liberated form then enters the nucleus to trigger the transcription of target genes essential for lipid biosynthesis, such as ATP citrate lyase (*ACLY*), acetyl-CoA carboxylase (*ACACA*), fatty acid synthase (*FASN*), and sterol-CoA desaturase (*SCD*) [9].

Quizartinib (AC220), a representative second-generation FLT3 inhibitor, was developed to target FLT3 with high specificity. Thus, quizartinib is more selective with less off-target toxicity and higher potency compared to the first-generation inhibitors [10]. Quizartinib has recently been approved by FDA for the combination treatment with standard cytarabine and anthracycline induction and consolidation therapy and as maintenance monotherapy following consolidation chemotherapy for the treatment of adult patients with newly diagnosed AML with FLT3/ITD mutation [11]. As monotherapy, FLT3 inhibitors was initially limited due to incomplete or transient clinical responses and primary or acquired resistance [12]. It is important to further delineate the role of FLT3 inhibitors on the mechanism of its interaction with the FLT3-mutated cells in order to optimize its therapeutic potential.

In this study, we investigated the regulation of FLT3/ITD mutation on SREBP and the therapeutic vulnerabilities of fatty acid metabolism in FLT3/ITD leukemia. We demonstrate that FLT3/ITD promotes the stability of SREBP via suppression of its protein degradation and show that inhibition of FLT3/ITD by quizartinib causes disruption of membrane lipid prior to cell death. Importantly, this metabolic dependency on membrane lipid homeostasis can be exploited to sensitize FLT3/ITD AML cells to quizartinib treatment. We examined the combinatorial approaches using fatostatin, which hinders the activation of SREBPs [13], and orlistat, a FASN inhibitor [14]. Our murine FLT3/ITD AML model showed a promising survival benefit provided by combination of fatostatin/quizartinib or orlistat/quizartinib over monotherapies. Collectively, these data reveal key insights into metabolic reprogramming events driven by FLT3 mutations in AML and suggest a novel combinatorial therapeutic strategy to enhance the targeted therapy of this aggressive subtype of AML.

## Results

### An active lipid metabolism is associated with worse AML prognosis and regulated by FLT3/ITD

**Fig. 1:**
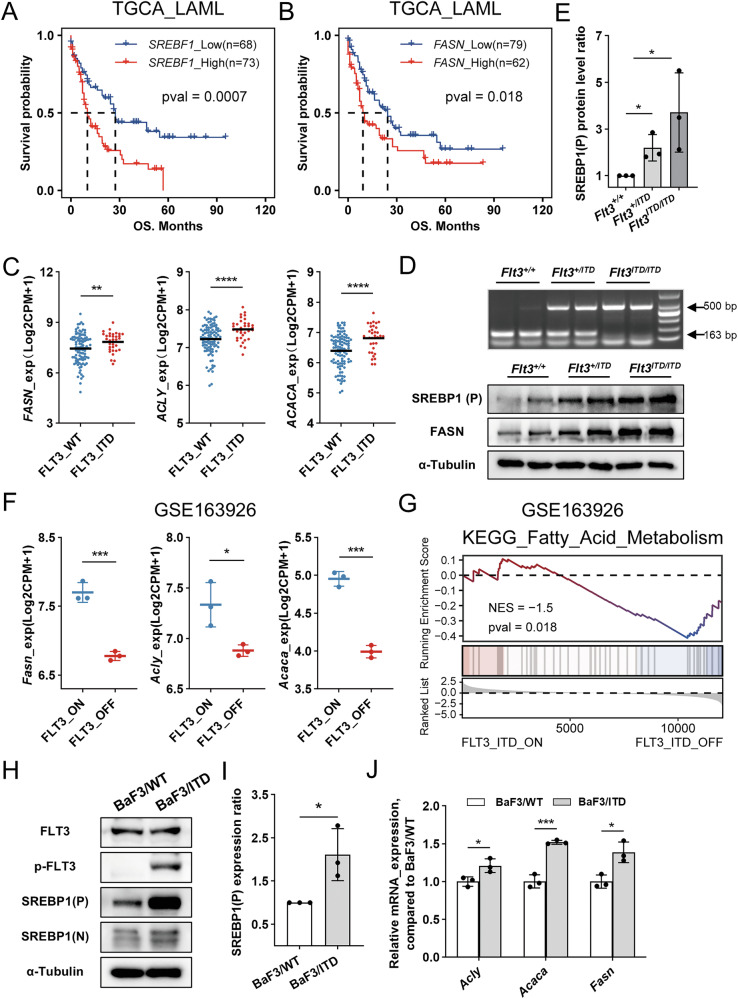
Upregulated lipid metabolism in FLT3/ITD leukemia. **A**, **B** Association between overall survival of AML patients and *SREBF1/FASN* expression in the TCGA_LAML cohort. **C** Normalized log2 counts-per-million (CPM) gene-expression values of *FASN*, *ACLY* and *ACACA* in FLT3/WT (*n* = 103) and FLT3/ITD (*n* = 34) patients from the TCGA_LAML cohort. **D** Upper panel, PCR analysis of mouse ear DNA from *Flt3*^+/+^(FLT3/wild type), *Flt3*^*+/ITD*^ (FLT3 heterogeneous), and *Flt3*^*ITD/ITD*^ (FLT3 homogeneous) animals. 163 bp, FLT3/wild type gene fragment; 500 bp, FLT3/ITD gene fragment. Lower panel, western blotting of SREBP1 (precursor form) and FASN expression in splenocytes of *Flt3*^*+/+*^, *Flt3*^*+/ITD*^, and *Flt3*^*ITD/ITD*^ mice. Each lane represents one mouse. **E** Comparison of SREBP1 protein expression levels in *Flt3*^*+/+*^, *Flt3*^*+/ITD*^, and *Flt3*^*ITD/ITD*^ mice. *N* = 3 replicates. Bars, means ± S.D. **F** Normalized log2CPM gene-expression of *Acly*, *Acaca* and *Fasn* in FLT3/ITD-ON vs. FLT3/ITD-OFF cells from GSE163926 dataset. Bars, means ± S.D., *n* = 3 per group. **G** Gene set enrichment analysis (GSEA) using the KEGG signature for fatty acid metabolism (KEGG ID: mmu01212) in the GSE163926 dataset. NES normalized enrichment score. **H** Activation of FLT3/ITD and its effect on SREBP1 expression was detected in BaF3/WT and BaF3/ITD cells by wester blotting analysis. P precursor form, N nuclear form. α-Tubulin was used as loading control. **I** Comparison of SREBP1 protein expression levels in BaF3/WT and BaF3/ITD cells. *N* = 3 replicates. Bars, means ± S.D. **J** Real-time quantitative PCR analysis showing upregulation of mRNA level of SREBP1-target genes in BaF3/ITD cells compared to the BaF3/WT cells.

We first analyzed The Cancer Genome Atlas (TCGA) LAML (acute myeloid leukemia) dataset and found that higher levels of *SREBF1* expression correlated with significantly worse patient outcome (*p* = 0.0007) in acute myeloid leukemia (Fig. 1A). High levels of SREBP1 target gene set such as *FASN* (*p* = 0.018) and *ACLY* (*p* = 0.0027) which are key enzymes for fatty acid synthesis were consistently associated with lower survival probability (Fig. 1B and Supplementary Fig. 1). Meanwhile, *SREBF2*, the master regulator of cholesterol synthesis, and its target genes responsible for cholesterol biosynthesis such as *LSS* and *SQLE* also exhibited an inverse correlation with AML patient survival (Supplementary Fig. 2A). Genes involved in fatty acid synthesis were particularly higher in FLT3/ITD patients compared with FLT3 wild type patients (TCGA_LAML cohort), including *FASN*, *ACLY* and *ACACA* (Fig. 1C). Genes involved in cholesterol metabolism including *DHCR7*, *SQLE* and *LSS* were also upregulated in patients with FLT3/ITD mutation (Supplementary Fig. 2B). A genetically engineered mouse model [15] was further used to investigate the alteration in lipid metabolism in AML harboring FLT3/ITD mutation. As shown in Fig. 1D, both heterozygous (*Flt3*^*+/ITD*^) and homozygous (*Flt3*^*ITD/ITD*^) animals carried mutant ITD alleles. Compared with the wild type (*Flt3*^+/+^), *Flt3*^*ITD/ITD*^ mice demonstrated increased protein levels of both SREBP1 and FASN. Such increase was also observed in *Flt3*^*+/ITD*^ mice to a lesser degree (Fig. 1D, E), indicating that FLT3/ITD mutation may activate the SREBP-mediated lipid metabolism pathway. We further examined the GSE163926 dataset from another mouse model of doxycycline inducible FLT3-ITD expression in MLL-AF9–rearranged AML [5]. As shown in Fig. 1F and Supplementary Fig. 2C, depletion of FLT3 in the MLL-AF9/iFLT3-ITD cells caused significant decrease of SREBP target genes in both fatty acid and cholesterol metabolism pathways. Gene set enrichment analysis (GSEA) consistently showed suppression of “fatty acid metabolism” in the KEGG pathway gene sets (Fig. 1G). We then ectopically expressed FLT3/wild type and FLT3/ITD in BaF3 cells to further evaluate the effect of oncogenic activation of FLT3/ITD on SREBP expression. Figure 1H showed abundant FLT3 protein expression in both FLT3/wild type and FLT3/ITD cell lines. However, FLT3/ITD mutation caused elevation of phospho-FLT3, the precursor and nuclear form of SREBP1 compared with the wild type (Fig. 1H, I). The mRNA expression of SREBP1 target genes including *Acly*, *Acaca*, and *Fasn* was also upregulated in BaF3/ITD cells (Fig. 1J). Our results indicates that FLT3/ITD mutation may have a direct impact on SREBP1 expression through the activated downstream cascade of FLT3 tyrosine kinase.

### Pharmacological inhibition of FLT3/ITD causes suppression of SREBP target genes

**Fig. 2:**
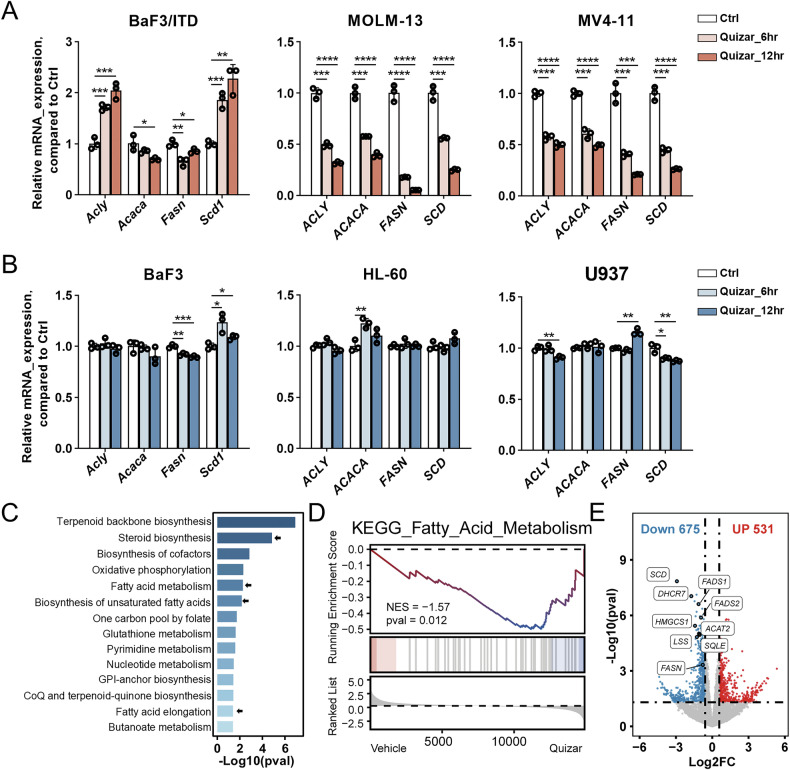
Quizartinib inhibits SREBP target gene expression in FLT3/ITD leukemia cells. Relative mRNA levels of major enzymes in the fatty acid synthesis pathway from FLT3-ITD cell lines (**A**) and FLT3-WT cell lines (**B**). Ctrl, control cells without treatment. Quizar_6 h, treatment with quizartinib (10 nM) for 6 h. Quizar_12 h, treatment with quizartinib (10 nM) for 12 h. Bars, means ± SD, *n* = 3 per group. **C** KEGG metabolism related pathways of downregulated genes in CD45 positive cells after quizartinib treatment in GSE202222 dataset. Arrows, lipid metabolism pathways. **D** Gene set enrichment analysis of the KEGG signature fatty acid metabolism (KEGG ID: hsa01212) after quizartinib treatment in GSE202222 dataset. **E** Volcano plot of differentially expressed genes in GSE202222. Labeled genes were those downregulated in the fatty acid or lipid metabolism after quizartinib treatment (*p* < 0.05, |Fold Change| > 1.5).

To test if pharmaceutical inhibition of FLT3/ITD also perturb the SREBP-mediated fatty acid metabolism as the genetic depletion, quizartinib, a highly specific inhibitor with potent binding affinity for FLT3 [16, 17] was used to treat the murine and human cell lines with FLT3/ITD mutation. Quizartinib consistently caused suppression of mRNA expression of *ACLY*, *ACACA*, *FASN*, and *SCD* in a time dependent manner from 6 to 12 h in the human MOLM-13 and MV4-11 cells (Fig. 2A). Interestingly, in the murine BaF3/ITD cells, we only observed decrease in *Acaca* and *Fasn*, but not in *Acly* and *Scd1*. Previous studies suggest that the target genes of SREBPs are cell type-specific and tissue-specific [6]. We reasoned that the observation may reflect the diverse role of SREBP in gene regulation across different contexts. Furthermore, the same treatment caused minimum effect on the above genes in cells harboring FLT3 wild-type including BaF3, HL-60, and U937 (Fig. 2B). To explore the broader implications of these findings, we further investigated the GSE202222 dataset from a xenograft mouse model engrafted with primary human FLT3/ITD AML cells and treated with quizartinib for 16 h [18]. Metabolic pathway analysis revealed the significant suppression of major lipid metabolism following treatment of quizartinib including “steroid biosynthesis”, “fatty acid metabolism”, “biosynthesis of unsaturated fatty acids”, and “fatty acid elongation” (Fig. 2C). GSEA analysis confirmed significant suppression of “fatty acid metabolism” in the KEGG pathway gene sets (*p* = 0.012) (Fig. 2D). Among the most suppressed genes subsequent to quizartinib treatment in vivo, we identified genes involved in fatty acid metabolism including *FASN* and *SCD*, and genes involved in cholesterol metabolism including *DHCR7*, *LSS*, *SQLE* and *HMGCS1* (Fig. 2E). The suppression of the genes in fatty acid metabolism appeared to be consistent with our observation in the cell line models with quizartinib treatment. Collectively, these data suggest that FLT3/ITD may control lipid metabolism via transcriptional mechanism regulated by SREBP.

### FLT3/ITD plays an important role in regulating SREBP degradation and stability via AKT/GSK3β axis

**Fig. 3:**
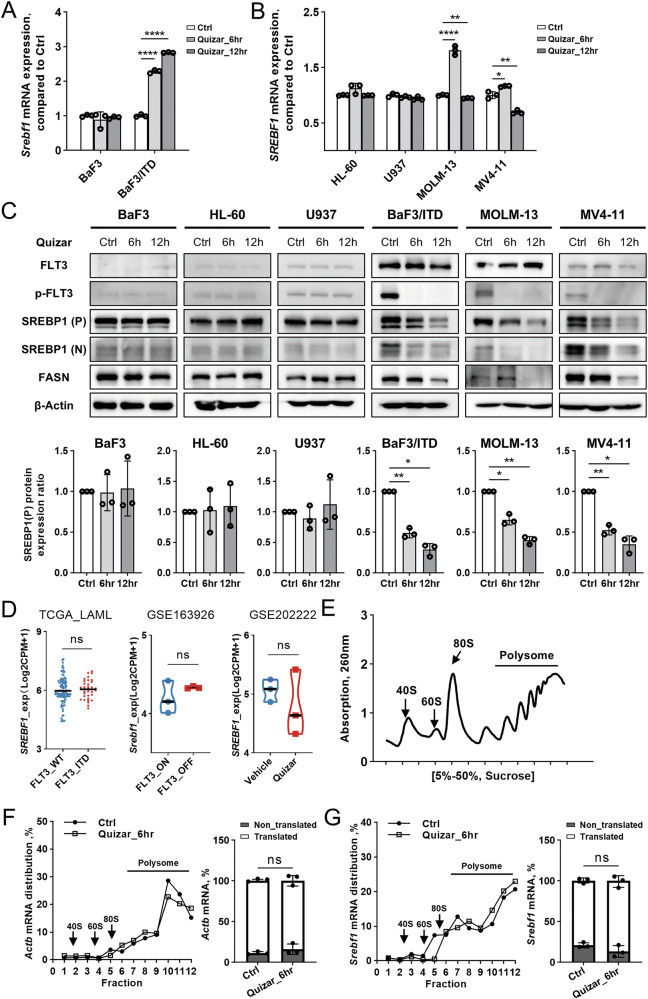
Quizartinib suppresses protein expression of SREBP1 in FLT3/ITD leukemia cells. Relative *Srebf1*/*SREBF1* mRNA level in murine (**A**) and human (**B**) leukemia cell lines with control, quizartinib (10 nM) treatment for 6 h and 12 h. *N* = 3 replicates. Bars, means ± S.D. **C** Upper panel, western blotting of SREBP1 and FASN in FLT3-WT and FLT3-ITD cell lines before and after quizartinib treatment of 6 and 12 h. P precursor form, N nuclear form. Lower panel, comparison of SREBP1 protein expression in the FLT3/ITD cells before and after quizartinib treatment. *N* = 3 replicates. Bars, means ± S.D. **D** Log2CPM gene-expression values of *SREBF1*/*Srebf1* in TCGA_LAML, GSE163926 and GSE202222 datasets. **E** The polysome profile of BaF3/ITD cells. 40S, 60S, and 80S ribosomal subunits and polysomes were fractionated using 5–50% density gradient sucrose buffer and monitored with A260 measurements. **F**, **G** Total RNA of BaF3/ITD cells before and after quizartinib treatment was extracted from each fraction. The expression of *Srebf1* and *Actb* was measured by qPCR. The mRNA content from monosomes (40S, 60S, 80S) represent the non-translated portion. The mRNA content from polysome represents the translated portion. *N* = 3 independent experiments. Bars, means ± S.D.

We next sought to investigate the molecular mechanism underlying the gene regulation involved in lipid metabolism by FLT3/ITD. We first examined the expression level of *SREBF1*, which encodes the central transcription factor SREBP1 for lipid metabolism. We observed a significant upregulation in the mRNA levels of *SREBF1* following a 6-h quizartinib treatment in both murine (BaF3/ITD) and human (MOLM-13, MV4-11) cell lines with FLT3/ITD mutation (Fig. 3A, B). The increase subsequently declined to levels comparable to the control group in the human cell lines after 12 h (Fig. 3B). Nevertheless, the protein expression of both precursor and nuclear forms of SREBP1 exhibited a notable time-dependent decrease following treatment with quizartinib for 6 and 12 h in all FLT3/ITD mutated cell lines (Fig. 3C). In contrast, the same treatment of quizartinib had minimal effect on both mRNA and protein expression in FLT3/wild type cells (BaF3, HL-60 and U937) (Fig. 3A, B). Analysis of the TCGA_LAML, GSE163926, and GSE202222 datasets further suggested that the activation of FLT3/ITD mutation or inhibition of FLT3 did not exert a significant effect on the mRNA levels of *SREBF1* (Fig. 3D). We also observed inhibition of FASN protein expression in FLT3/ITD mutated cells following quizartinib treatment, which further indicated the metabolic alterations in lipid metabolism mediated by FLT3-SREBP-FASN axis (Fig. 3C).

Our results indicated that FLT3/ITD may play an important role in regulating SREBP expression at the protein level in leukemia cells. To test whether FLT3/ITD impact the translation of SREBP1, total RNA was extracted from monosome (40 and 60S), disome (80S), and polysome fractions of BaF3/ITD cells and separated over 5%–50% sucrose gradients and the distribution from each fraction was compared (Fig. 3E). β-Actin mRNA (*Actb*), used as a positive control, were highly translated with a great number of polysomes (peaks shift to the right), and equally associated with polysomes before and after quizartinib treatment (Fig. 3F). The *Srebf1* expression also demonstrated similar distribution among each fraction in both treatment conditions (Fig. 3G). Our results further showed that inhibition of FLT3 appear not to impede the protein translation of SREBP1.

**Fig. 4:**
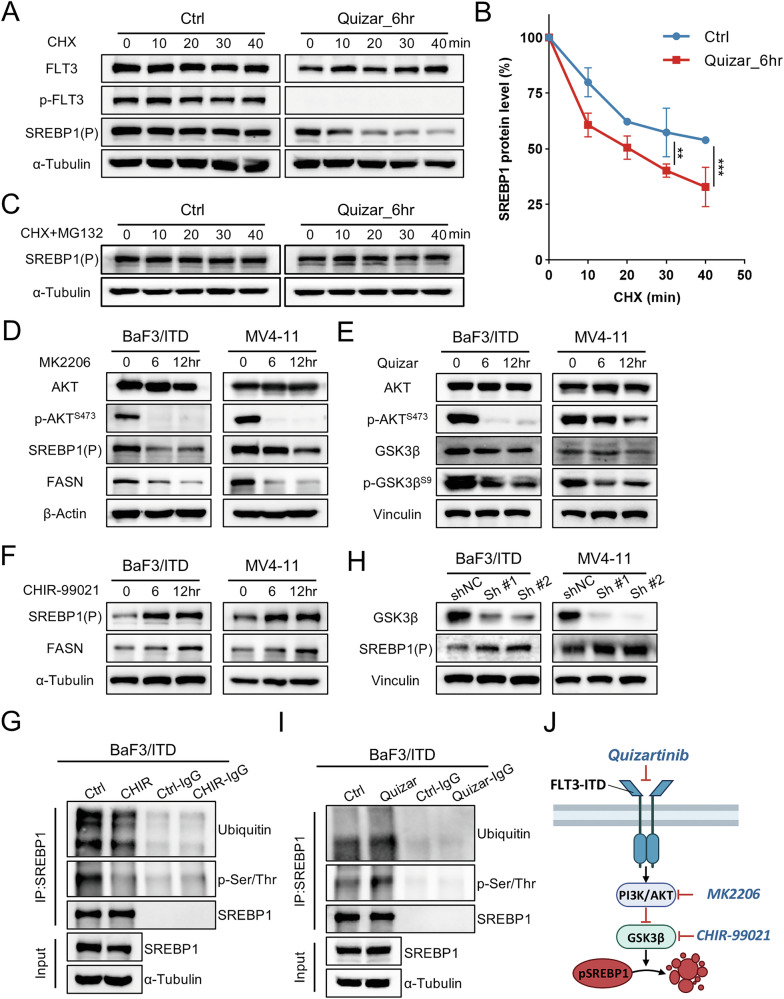
Quizartinib promotes protein degradation of SREBP1. **A** BaF3/ITD were pre-treated with vehicle control (DMSO) or quizartinib (10 nM) for 6 h. SREBP1 (precursor) protein expression was then detected after treatment with cycloheximide (CHX, 100 μg/mL) at the indicated time points. **B** The plotted graph of three independent experiments showing the relative amount of SREBP1 at each time point compared to the control without CHX treatment. **C** Western blotting showing SREBP1 protein expression in the presence of CHX (100 μg/mL) and MG132 (10 μM). **D** Expression of total AKT, phosphor-AKT (p-AKT^S473^), SREBP1 and FASN in BaF3/ITD and MV4-11 cells before and after treatment of MK2206 (5 μM) for 6 and 12 h. **E** Expression of total AKT, phospho-AKT (p-AKT^S473^), total GSK3β and phospho-GSK3β (p-GSK3β^S9^) in BaF3/ITD and MV4-11 cells before and after treatment of quizartinib (10 nM) for 6 and 12 h. Vinculin was used as loading control. **F** Expression of SREBP1 and FASN in BaF3/ITD and MV4-11 cells before and after treatment of CHIR-99021 (3 μM) for 6 and 12 h. **G** The ubiquitination and phosphorylation (pan phospho-Ser/Thr) levels of SREBP1 before and after treatment of CHIR-99021 for 6 h. IP immunoprecipitation. Total input of SREBP1 and α-Tubulin was shown as equal loading. **H** Expression of SREBP1in BaF3/ITD and MV4-11 cells with shRNA targeting *GSK3β*. NC non-specific control. Sh #1 and Sh #2, different sequencing of shRNA targeting *GSK3β*. **I** The ubiquitination and phosphorylation (pan phospho-Ser/Thr) levels of SREBP1 before and after treatment of quizartinib for 6 h. **J** An illustrated pathway displaying the regulation of protein degradation of SREBP by the FLT3/AKT/GSKβ phosphorylation cascade and the kinase inhibitors including quizartinib, MK2206 and CHIR-99021.

Next, we examined the SREBP1 degradation pathway in FLT3/ITD mutated cells. It is known that SREBPs are activated by two sequential cleavage steps in Golgi and are transported into the nucleus. Previous studies showed that not only nuclear SREBPs but also their precursor forms are subjected to degradation by the ubiquitin proteasomal system [19, 20]. We used the proteasome degradation pathway inhibitor MG132 to test if the proteasomal pathway is involved in FLT3/ITD-mediated SREBP degradation. BaF3/ITD cells were first treated with cycloheximide (CHX) for 10–40 min to prevent new protein synthesis. Western blotting analysis showed a progressive decrease in the precursor form of SREBP1 from 10–40 min after CHX treatment in the BaF3/ITD cells. In comparison, the turnover of SREBP protein was significantly promoted when cells were pretreated with quizartinib (Fig. 4A, B). We then examined whether inhibition of protein degradation is able to prevent such decrease caused by quizartinib. Figure 4C showed that treatment with MG132 for 10–40 min restored the SREBP protein levels of quizartinib-treated cells even in the presence of CHX. These data suggest that FLT3/ITD may enhance the stabilization of SREBP via regulation of its degradation.

It is known that oncogenic signaling induces an increased dependence on de novo lipogenesis to fulfill the bioenergetic and biosynthetic demands of rapidly proliferating cells. The FLT3-PI3K-AKT signaling is an established pathway that is constitutively activated in FLT3/ITD acute myeloid leukemia [21]. It has also been shown that ubiquitination can be preceded by phosphorylation of nuclear form SREBP1 by glycogen synthase kinase‑3β (GSK3β). Activation of GSK-3 resulted in enhanced degradation of SREBP1 [22, 23]. We reasoned that FLT3/ITD may promote the stability of SREBP1 by a phosphorylation cascade in the AKT/GSK3β axis. Indeed, MK2206, a selective inhibitor of AKT, caused decrease of SREBP1 in both BaF3/ITD and MV4-11 cells as early as 6 and 12 h (Fig. 4D). The inhibition of FLT3 by quizartinib suppressed the phosphorylation of both AKT and GSK-3β (Fig. 4E). CHIR-99021, a highly specific GSK-3 inhibitor reduced the phosphorylation and ubiquitination of SREBP1, meanwhile promoted the accumulation of SREBP1 as early as 6 h (Fig. 4F, G). Consistent with the effect of pharmacological inhibition, knockdown of *GSK3β* by shRNA also led to the accumulation of SREBP1(Fig. 4H). Finally, quizartinib caused concomitant increase of phosphorylation and ubiquitination of SREBP1 (Fig. 4I). The statistical analysis of western blots in FLT3/AKT/GSK3/SREBP1 axis was shown in Supplementary Fig. 3. To further verify the regulation of SREBP1 activity by FLT3/ITD, we performed subcellular localization analysis of SREBP1. We found that quizartinib treatment reduced SREBP1 expression in both cytosolic (precursor form) and nuclear (active form) fractions. Conversely, CHIR-99021 treatment increased SREBP1 expression in both fractions (Supplementary Fig. 4). As summarized in Fig. 4J, our results suggest that oncogenic activation of FLT3/ITD can stabilize the SREBP1 protein through AKT by inhibiting its negative regulator, GSK3β.

### FLT3-ITD regulates global lipid metabolism and maintains mitochondrial membrane homeostasis

**Fig. 5:**
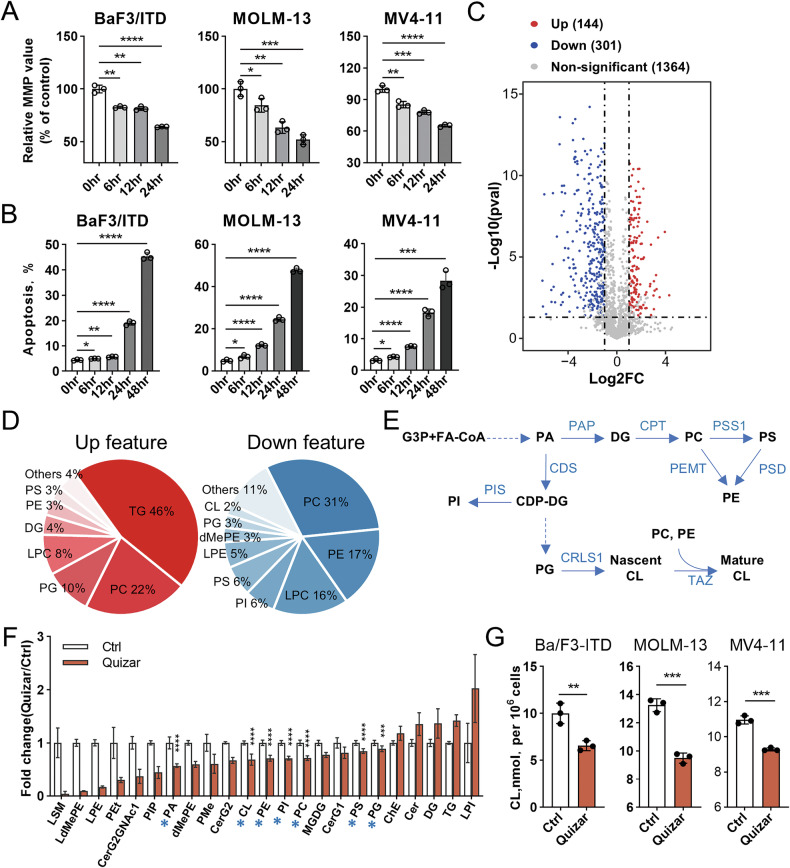
Quizartinib suppresses phospholipid metabolism in FLT3/ITD leukemia cells. **A**, **B** The FLT3-ITD leukemia cell lines were treated with vehicle or quizartinib (10 nM) at the indicated time points. Mitochondrial membrane potential and cell death was assessed by Rho123 and Annexin V/PI, respectively. *n* = 3 per group. Bars, means ± S.D. **C** Lipidomic analysis of MOLM-13 cells before and after treatment of quizartinib (10 nM) for 12 h. Significant differentially abundant metabolites between two groups (*p* < 0.05, |Log2FC | > 1 and VIP > 1) are shown in red (upregulated) and blue (downregulated) in the volcano plot. **D** The pie graph illustrating the proportion of each lipid class contributing to the total number of upregulated (red) and downregulated (blue) features. **E** The illustration of enzymatic steps involved in biosynthesis of major phospholipids. **F** Comparison of lipid abundance between the control and treatment group in all lipid classes with statistical significance. Blue stars, major types of phospholipids involved in the biosynthesis pathway. *N* = 6 replicates. Bars, means ± S.D. ***(*p* < 0.001) and ****(*p* < 0.0001) indicate *p* values for the major phospholipids with blue stars. *p* < 0.05 for all other lipid species. **G** The absolute cardiolipin contents in FLT3/ITD cells treated before and after quizartinib (10 nM, 12 h) were detected by the fluorometric assay as described in the method. *N* = 3 replicates. Bars, means ± S.D. The abbreviations of all lipid species and enzymes were listed in the Supplementary Table 1.

We next investigated the downstream metabolic consequences of FLT3/ITD inhibition. Quizartinib treatment reduced the mitochondrial membrane potential in a time dependent manner (6–24 h) in both murine and human cells with FLT3/ITD mutation (Fig. 5A and Supplementary Fig. 5A). The loss of membrane potential caused subsequent induction of apoptosis in these cells (Fig. 5B and Supplementary Fig. 5B). The same treatment did not cause any detectable effect in the transmembrane potential or cell death in the FLT3/wild-type cells (Supplementary Fig. 5A, B). It is known that phospholipids are the building blocks that make up the characteristic mitochondrial structures such as the cristate, matrix, outer mitochondrial membrane (OMM) and the inner mitochondrial membrane (IMM) [24]. Lipidomics was performed on FLT3/ITD-mutated cells before and after treatment by quizartinib and the number of lipid species detected in the MOLM-13 cells was shown in Supplementary Fig. 6A. We found 144 upregulated and 301 downregulated lipid features, respectively, in MOLM-13 cells after treatment with quizartinib (*p* < 0.05, |Log2FC| > 1 and VIP > 1) (Fig. 5C).

Further analysis of lipid classes with different compositions demonstrated that inhibition of FLT3 caused significant reduction of phospholipid and elevation of diglycerides (DGs) and triglycerides (TGs). Phospholipid, constituted approximately 90% of the significantly altered lipids with downregulation. Meanwhile, diacylglycerols (DGs) and triacylglycerols (TGs) comprised approximately 50% of the most differentially altered lipids with upregulation (Fig. 5D). Figure 5E illustrated the pathway for de novo synthesis of major phospholipids. The abbreviation of the major lipid species and enzymes were shown in Supplementary Table 1. It is known that phosphatidylcholine (PC) and phosphatidylethanolamine (PE) are the most abundant phospholipids in mammalian cell membranes. PC and PE are also essential acyl chain donors for the synthesis of the mature form of the mitochondrial-specific phospholipid cardiolipin [25]. Unsupervised hierarchical clustering separated cells treated with quizartinib from the control cells, based on the distinct profiles of top 12 significantly altered lipid species (*p* < 0.05) (Supplementary Fig. 6B). The lipidomics analysis of all lipid species demonstrated the significant downregulation (*p* < 0.05) of major phospholipids in this pathway including PA, PC, PS, PE, PG, PI and CL, and significant upregulation (*p* < 0.05) of DG and TG (Fig. 5F). Cardiolipin plays an essential role in mitochondrial structure, bioenergetics and apoptosis [26]. Finally, by measuring the absolute levels, we confirmed the decrease of cardiolipin in different FLT3/ITD mutated cells lines after treatment of quizartinib (Fig. 5G). We further tested whether FLT3/ITD may regulate the mitochondrial function such as biogenesis. Here, the fluorescent probe MitoTracker green was used to detect the mitochondria number. As shown in Supplementary Fig. 7, inhibition of FLT3/ITD by quizartinib caused no detectable change of MitoTracker green level in all FLT3/ITD cells. As such, inhibition of lipid biosynthesis may be a metabolic vulnerability in FLT3/ITD leukemia cells and could be exploited for treatment of this subtype of leukemia.

### Blocking lipid synthesis enhances the therapeutic efficacy of quizartinib in vitro and in vivo

**Fig. 6:**
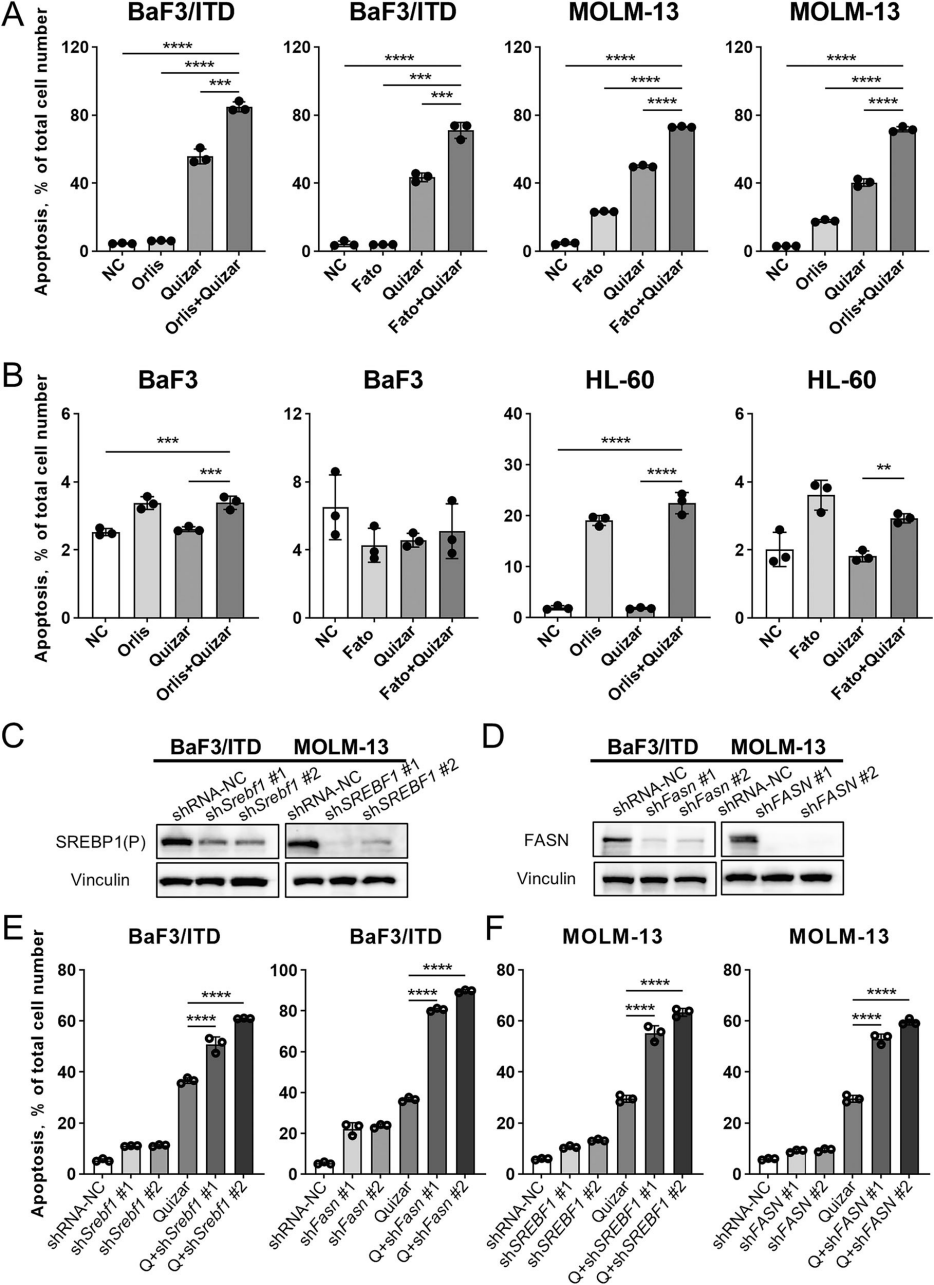
Inhibition of SREBP and FASN enhances quizartinib induced cell death in FLT3/ITD leukemia cells. **A**, **B** Quantitative analysis of cell death after different drug treatment for 48 h in FLT3/ITD (BaF3/ITD, MOLM-13) and FLT3-WT (BaF3, HL-60) cells. *N* = 3 replicates. Bars, means ± S.D. Ctrl control cells treated with vehicle (DMSO), Orlis orlistat (10 μM), Quizar quizartinib (10 nM), Fato fatostatin (5 μM). **C**, **D** Western blot analysis showing knockdown of SREBP1 and FASN protein in BaF3/ITD and MOLM-13 cells by shRNA. NC non-specific shRNA sequence. **E**, **F** Quantitative analysis of cell death in BaF3/ITD and MOLM-13 cells following quizartinib treatment, with or without SREBP and FASN knockdown by shRNA.

Given our findings that FLT3 inhibition caused decrease of phospholipid levels and subsequent loss of mitochondrial transmembrane potential, we hypothesized that blockade of the SREBP pathway and inhibition of FLT3 would exacerbate disruption of membrane homeostasis driven by FLT3/ITD. Here, fatostain and orlistat were used to block the SREBP activation and FASN, respectively. Fatostatin is a small molecule that inhibits the ER-Golgi translocation of SREBPs by binding to the escort protein SCAP [13]. Orlistat is an irreversible inhibitor of FASN via the thioesterase (TE) domain of the enzyme [14]. As shown in Fig. 6A, treatment of 10 nM quizartinib alone for 48 h caused 40–50% cell death in BaF3/ITD cells as measured by Annexin-V/PI assay. Treatment of 5 μM fatostatin or 10 μM orlistat alone caused minimum cytotoxic effect in BaF3/ITD cells. However, combination of quizartinib and fatostatin/orlistat substantially enhance the cell death to more than 70–80%. The combination effect was also confirmed in human FLT3/ITD cell line MOLM-13 (Fig. 6A). In contrast, the combination of FLT3 inhibitor and perturbation of SREBP pathway did not induce any combination effect in FLT3-wild type cell lines (BaF3 and HL-60) (Fig. 6B). We further used shRNA to knock down SREBP and FASN (Fig. 6C, D) to test the combination effect. Indeed, silencing of SREBP and FASN consistently enhanced the cytotoxic effect of quizartinib in FLT3-mutated cells (Fig. 6E, F). The flow cytometry analysis of apoptotic cell death induced by pharmacological inhibitors and gene silencing were shown in Supplementary Fig. 8.

SREBP-1 and SREBP-2 play distinct roles in lipid metabolism, with SREBP-1 primarily driving fatty acid synthesis and SREBP-2 regulating cholesterol biosynthesis. To further explore the impact of cholesterol metabolism on quizartinib sensitivity, we used statins to inhibit HMG-CoA reductase, a key enzyme in the mevalonate pathway. However, the combination of statins and quizartinib demonstrated an antagonistic effect in FLT3/ITD cells (Supplementary Fig. 9). Therefore, our data suggests that targeting the oncogenic activation of FLT3/SREBP/FASN axis may be a more effective strategy to enhance the anti-leukemia effect of quizartinib.

**Fig. 7:**
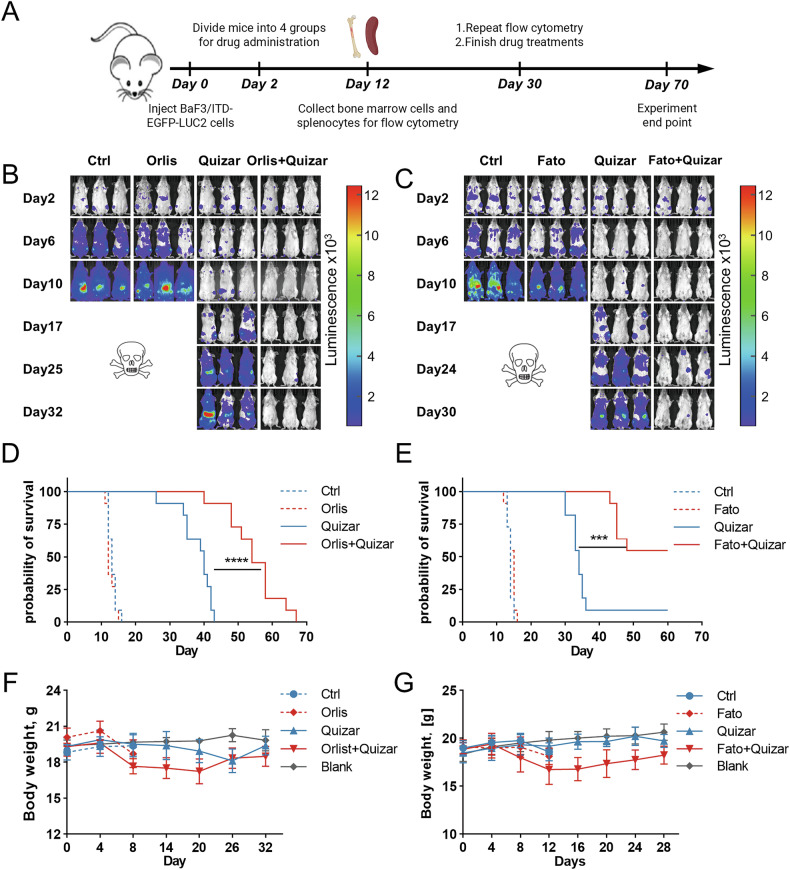
Pharmacological inhibition of SREBP and FASN enhances the therapeutic effect of quizartinib in a murine FLT3/ITD leukemia model. **A** Illustration of the animal experiment: 2 days after tail vein injection of BaF3/ITD-EGFP-LUC2 cells, BALB/c mice were randomized and treated with fatostatin (15 mg/kg i.p. daily), orlistat (240 mg/kg i.p. daily), quizartinib (1 mg/kg oral daily), or quizartinib plus fatostatin/orlistat for another 28 days. **B**, **C** Leukemia burden and progression was monitored by luminescence imaging every 4 days at early stage and every week at late stage. *n* = 3 mice per group. **D**, **E** Survival was estimated by Kaplan–Meier analysis as described in Methods. *n* = 11 mice in each group. **F**, **G** Measurement of body weights of each group. Blank normal mice without leukemia cell injection, Ctrl control group with leukemia burden, Orlis orlistat, Fato fatostatin, Quizar quizartinib.

We next sought to determine whether fatostatin or orlistat could enhance the anti-leukemia activity of FLT3 tyrosine kinase inhibitor in vivo. We conducted two independent experiments to test the combination effect of quizartinib plus fatostatin or orlistat. BALB/c mice were transplanted with GFP/luciferase-bearing BaF3/ITD cells by tail injection. On Day 2 after leukemia transplantation, mice were randomized and treated with fatostatin, orlistat, quizartinib, quizartinib in combination with fatostatin or orlistat, or empty solvent as the control (Fig. 7A). As the bioluminescence shown in Fig. 7B, C, the control and fatostatin/orlistat treated mice demonstrated progressive leukemia burden around Day 10. It appeared that fatostatin or orlistat alone had negligible effect in vivo. Mice treated with quizartinib alone delayed the burden progression and relapsed at a later time point around Day 17. In comparison, fatostatin or orlistat combined with quizartinib appeared to resist the leukemia progression. On Day 30 when drug treatment ended, the leukemia-derived fluorescence in bone marrow demonstrated that mice treated with fatostatin plus quizartinib had significantly lower leukemia burden than did quizartinib-treated mice (*p* < 0.05; Supplementary Fig. 10A, B). The leukemia infiltration in spleen also confirmed the similar result (Supplementary Fig. 10C, D). Most importantly, orlistat plus quizartinib significantly prolonged the median survival by 35%, from 40 days in the group treated with quizartinib alone to 54 days (Fig. 7D). Furthermore, 6 out of 11 animals survived in the group treated with fatostatin plus quizartinib 40 days after drug administration ended. In comparison, only 1 out 11 animals survived in the group treated with quizartinib alone (Fig. 7E). As both fatostatin and orlistat are compounds that interfere with the lipid synthesis pathway, we closely monitored changes in body weight of the treated mice. As shown in Fig. 7F, G, the animals exhibited weight loss from Day 8 to Day 20 when treated with quizartinib plus fatostatin or orlistat. However, their body weights climbed back at the later time points paralleled to the level comparable to the those treated with quizartinib alone. We further evaluated the combined effect of fatostatin and quizartinib on lipid metabolism in vivo using *Flt3*^*ITD/ITD*^ mice. The mice were treated with fatostatin, quizartinib, or the combination of both compounds. As shown in Supplementary Fig. 11, compared to the single agent alone, the combination treatment significantly reduced lipid content in FLT3/ITD-mutated cells from the transgenic mice (*p* < 0.05). The above results demonstrate that pharmacological inhibition of SREBP pathway provides a significant therapeutic benefit over FLT3 tyrosine kinase inhibitors alone and may be tolerated by normal cells.

## Discussion

Recent studies have demonstrated the metabolic alterations associated with FLT3 mutation in leukemia cells. We previously reported that FLT3/ITD mutation promotes Warburg effect and renders therapeutic sensitivity to inhibition of glycolysis [2]. Bjelosevic et al. reported that FLT3/ITD promotes serine synthesis and uptake via ATF4-dependent transcriptional regulation of genes in the de novo serine biosynthesis pathway [5]. It has also been shown that glutamine metabolism, through its ability to support mitochondrial function and cellular redox metabolism, becomes a metabolic dependency of FLT3/ITD AML [3]. However, the functional importance of lipid metabolism and therapeutic implications in FLT3-mutant AML has remained to be further explored. Sterol regulatory element-binding protein (SREBP) is a well-established transcription factor to promote de novo lipogenesis [6]. The role of SREBP and its regulation in leukemia, particularly, over the course of oncogenic transformation by FLT3/ITD is less well understood. SREBP1 is encoded by *SREBF1* gene [27]. In this study, we showed that higher levels of *SREBF1* and its target genes in the fatty acid synthesis pathway correlate with significant worse patient outcome. *ACLY*, *ACACA* and *FASN* expressions are particularly higher in patients with FLT3/ITD compared to FLT3/wild-type, suggesting the critical role of SREBP-mediated lipogenesis in this AML subtype. Interestingly, the analysis of TCGA-LAML and other datasets (GSE163926, GSE202222) showed that there is no significant alteration in mRNA expression of *SREBF1* when FLT3/ITD is activated. We reasoned that the oncogenic activation of FLT3 tyrosine kinase may play an important role in the regulation of SREBP stability.

SREBP resides in the endoplasmic reticulum as an inactive precursor and undergoes two sequential cleavage steps in the Golgi prior to translocation to the nucleus as a mature transcription factor. Both nuclear and precursor forms of SREBP are subjected to degradation by the ubiquitin proteasome pathway [19, 20]. In our study, we observed an accumulation of precursor form of SREBP1 protein in *Flt3*^*ITD/ITD*^ and *Flt3*^*+/ITD*^ mice compared with the wild type. The ratio of mutated FLT3 alleles to wild-type FLT3 alleles is often associated with more aggressive disease and poorer outcomes [21]. A noteworthy increase of SREBP1 in *Flt3*^*ITD/ITD*^ compared with FLT3^+/ITD^ mice was also detected, which may further support the role of FLT3/ITD activation in the signaling pathway for SREBP regulation. It has been shown in a number of cell lines that glycogen synthase kinase 3 (GSK3) is able to phosphorylate the consensus phosphopeptide motif (CPD) of SREBP, which further allows stimulation of its ubiquitination and proteasomal degradation [22, 28]. Meanwhile, PI3K/AKT, the established downstream effector of FLT3 tyrosine kinase, phosphorylates and inhibits GSK3 when activated [29]. Here, we showed that pharmacological inhibition of FLT3 tyrosine kinase not only caused decrease of protein expression of SREBP but also enhanced its phosphorylation. Our results indicates that FLT3/ITD may control the stability of SREBP through the phosphorylation cascade of PI3K/AKT/GSK3 axis. In relation to our current finding, a study has recently shown the association between C/EBPα-regulated lipid synthesis and FLT3/ITD mutation [30]. Interestingly, others suggested that LXR-C/EBP complex may bind to SREBP-1c promoter and increase mRNA expression in rat hepatocytes during insulin action [31]. It was reported that GSK3β induces C/EBP phosphorylation, which then promotes the expression of the *SREBF-1a* transcription factor during adipogenesis [32]. As such, the crosstalk between C/EBP and SREBP in the context of oncogenic activation of FLT3/ITD may be worth further investigation.

The accumulation of SREBP1 and FASN proteins in *Flt3*^*ITD/ITD*^ mice suggest the potential role of SREBP mediated lipid metabolism in the oncogenic transformation by FLT3/ITD mutation. While initial outcomes with monotherapy of FLT3 inhibitors were promising, the eventual emergence of resistance has remained a major challenge in the targeted therapy for this particularly aggressive subtype of AML [21]. One of our important findings in this study is that quizartinib caused rapid degradation of SREBP and specifically downregulated the levels of major species of phospholipids in FLT3/ITD leukemia cell lines. Taken together, these findings indicated a mechanistic link between modulation of SREBP mediated lipid metabolism and FLT3-targeted therapy of FLT3/ITD leukemia. Fatostatin has been identified as a diarylthiazole compound that disrupts the transport of SREBP-SCAP complex from endoplasmic reticulum to golgi apparatus, thereby suppressing SREBP activation [33]. Our results demonstrated that disrupting SREBP axis by fatostatin, and fatty acid synthase (FASN) inhibitor orlistat substantially enhanced the efficacy of quizartinib in vitro and in vivo. FASN is the sole enzyme capable of the de novo synthesis of long-chain fatty acids from acetyl-CoA and malonyl-CoA. Numerous studies have confirmed the potential of FASN as a target for anti-cancer therapy [34]. Orlistat, the FDA-approved for anti-obesity therapy, was initially developed as an inhibitor of pancreatic and gastric lipase. This compound has also demonstrated anti-cancer activities in various preclinical models as a potent FASN inhibitor [35, 36]. Due to the limited bioavailability in its current oral form for obesity, repurposing efforts have been aimed at formulating orlistat for systemic administration. Kridel et al. [14] reported that intraperitoneal injection of orlistat at 155 mg/kg in mice achieved peak blood levels around 10 μM, a concentration aligned with those used for cytotoxicity assay in the current study. Others suggested a dose of 240 mg/kg in mice, which also coincides with those used in our in vivo study, attained AUC concentrations above 25 μg/h/ml [35]. Taken together, our study offers a positive outlook on exploiting the specific lipid metabolism in the context of FLT3/ITD mutation. Our findings also provide a rationale for drug combination and clinical application in this subtype of leukemia.

## Materials and methods

### Cell lines and cell culture

The murine cell lines BaF3 and human cell lines MOLM-13, MV4-11, and U937 were purchased from DSMZ (German Collection of Microorganisms and Cell Cultures, Braunschweig, Germany). HL-60 were purchased from American Type Culture Collection (ATCC) (Manassas, VA, USA). All leukemia cell lines were cultured in RPMI 1640 medium (Gibco, Waltham, MA, USA) supplemented with 10% fetal bovine serum (FBS). BaF3 cells were additionally supplied with recombinant murine interleukin-3 (mIL-3, 10 ng/mL) (Public Protein/Plasmid Library, Nanjing, China). BaF3 cells were transfected with pSin4-EF2-IRES-Puro plasmid vector containing FLT3/wild type or FLT3/ITD fragments as previously described [15]. Transfected cells were cultured in RPMI 1640 containing 1 μg/mL puromycin (MP Biomedicals, Irvine, CA, USA) for selection. All cell lines in this study were authenticated by short-tandem repeat (STR) profiling and tested for mycoplasma contamination regularly.

### FLT3-ITD knock-in mice

The heterozygous FLT3/ITD Knock-in mice (*Flt3*^*+/ITD*^, B6.129-Flt3tm1Dgg/J with C57BL/6J background) were purchased from The Jackson Laboratories (West Grove, PA, USA). Heterozygous (*Flt3*^*+/ITD*^) mice were intercrossed to produce homozygous *Flt3*^*ITD/ITD*^ animals. The FLT3-ITD mutation was detected by PCR with primers as described in Supplementary Table 2. Splenocytes and bone marrow cells were collected from the mice for the immunoblotting analysis.

### Immunoprecipitation

BaF3/ITD cells were cultured and pretreated with quizartinib and MG-132 for 6 h. Cells were harvested and washed twice by pre-cold PBS. 5× IP lysis buffer (125 mM HEPES, 750 mM NaCl, 5 mM EDTA, 5% NP-40, 10% Glycerol, 1% protease inhibitor cocktail with pH = 7.4) was diluted by IP wash buffer (20 mM HEPES, 150 mM NaCl, 1 mM EDTA, 0.1% NP-40, 0.1% Glycerol with pH = 7.4) and added to cell pellets. The cells were vortexed thoroughly and sonicated for 3 times on ice (30 s on and 60 s off at 30 amplitude) followed by centrifugation at 12,000 rpm for 10 min. The supernatants were mixed with magnetic protein A/G (MCE) pre-incubated with mouse anti-SREBP1 antibody (1:100) or mouse anti-IgG for 6 h at 4 °C. The mixtures were incubated overnight at 4 °C and washed 5 times by IP wash buffer. Loading buffer was then added to magnetic beads before immunoblotting analysis.

### Nuclear-cytoplasmic separation

BaF3/ITD cells were cultured and pretreated with DMSO, quizartinib (10 nM) or CHIR-99021 (3 µM) for 6 h. Then cells were collected and washed twice by PBS. The cytoplasm and nucleus components were extracted by NE-PER Nuclear and Cytoplasmic Extraction kit (Thermo). The separated protein was stored at −80 °C for subsequent western blot analysis.

### Ribosome profiling

BaF3/ITD cells with or without quizartinib treatment were collected and incubated with CHX (100 µg/mL) (MCE) for 10 min to arrest translation. Cells were lysed with lysis buffer (10 mM Tris-HCl, pH = 7.4, 5 mM MgCl_2_, 100 mM KCl, 1% Triton X-100, 2 mM Dithiothreitol, 100 µg/mL CHX, 50 U/mL Rnase Inhibitor, 1 × protease Inhibitor) and centrifuged at 12,000 × *g* at 4 °C for 10 min. Supernatants were loaded onto the 5%–50% sucrose gradient solution prepared by Automated Gradient Maker System (BioComp, Canada) and centrifuged at 36,000 rpm for 2 h at 4 °C using ultra-high speed centrifuge (Beckman). The gradient fractions were separated and collected for absorption measurement at 260 nm. Total RNA from sucrose fractions was isolated by TRIzol reagent (Thermo) and subjected to real-time qPCR analysis. The sequences for *Srebf1* and *Actb* is described in Supplementary Table 2.

### Lipidomics analysis

A total of 1 × 10^7^ MOLM-13 cells (6 replicates per group) cells were collected and washed twice with pre-cold saline. A total of 225 μL frozen methanol was added to cell pellets and vortexed. A total of 750 μL pre-cold Methyl tert-Butyl (MTBE) was added to lysed cells and the mixture was vortexed on ice for 1 h at 400 rpm. A total of 188 μL MS-grade pure water containing internal standards was added to the sample and vortexed. The mixture was centrifuged at 15,000 × *g* at 4 °C for 10 min and the top layer containing lipids was transferred to a new tube. The sample was dried by Termovap Sample Concentrator and stored at −80 °C. Dried lipids were reconstituted in 120 μL mixture of isopropanol, methanol and water (60: 30: 5, v/v/v) and vortexed for 5 min before centrifuge at 15,000 × *g* at 4 °C for 10 min. The supernatant was transferred to a glass amber vial for the metabolomics study. The lipid analysis was performed on the ultra-performance liquid chromatography-high resolution mass spectrometry (UPLC–HRMS) platform which couples Dionex Ultimate 3000 UPLC system with Q-Exactive mass spectrometer (Thermo). Lipid Search v.4.1 was used for lipid annotation. Differential features were defined as *p* < 0.05 (the Student’s *t* test), |Log2FoldChange | > 1, and VIP value > 1 (Variable Important for the Projection, calculated by ropls package, v.1.34.0).

### Cardiolipin (CL) measurement

The Cardiolipin Assay Kit (Abcam) was used to measure cardiolipin content in cell lysates. The FLT3/ITD cell lines BaF3/ITD, MOLM-13 and MV4-11 were treated with quizartinib (10 nM) for 12 h. Cells were collected and washed twice. Pellets were suspended in CL assay buffer and lysed by sonicator. The lysates were reacted with fluorometric CL probes in 96-well plates for 5 min at room temperature and fluorescence value was measured at EX/EM 340/480 nm. The absolute cardiolipin content was calculated according to the standard curve.

### Animal experiment

All animal experiments were conducted following institutional guidelines approved by the Institutional Animals Care and Use Committee of Sun Yat-sen University Cancer Center. BaF3/ITD cells were transfected with plasmid pLenti-CMV-EGFP-LUC2-Hygro and 20 μg/mL hygromycin (InvivoGen, San Diego, CA, USA) was used for cell selection. A total of 5 × 10^5^ BaF3/ITD-EGFP-LUC2 cells were transplanted into the female BALB/c recipients (age 5–6 weeks) (GemPharmatech, Guangzhou, China) by tail vein injection. The mice were randomly divided into four groups (11 mice per group) 2 days after cell injection. The mice were then received treatment or vehicle control daily. The treatment of quizartinib and fatostatin was as the following: (1) Vehicle control (PBS containing 10% DMSO and 10% castor-oil); (2) 15 mg/kg fatostatin by intraperitoneal injection (dissolved in PBS containing 10% DMSO and 10% castor-oil); (3) 1 mg/kg quizartinib by oral gavage (dissolved in ddH_2_O) (MedChemExpress, Monmouth Junction, NJ, USA); (4) fatostatin + quizartinib. The treatment of quizartinib and orlistat was as the following: (1) Vehicle control by i.p. injection (50 μL volume with 33% ethanol and 66% PEG 400); (2) 240 mg/kg orlistat (Meilunbio, Dalian, China) via i.p. injection (dissolved in 50 µL volume containing 33% ethanol and 66% PEG 400); (3) 1 mg/kg quizartinib; (4) orlistat + quizartinib. The leukemia burden was measured by bioluminescence imaging every 4–7 days. The drug treatment ended on day 30 and the overall survival was monitored continuously until day 70. Mice were injected with 75 mg/kg D-luciferin (PerkinElmer, Waltham, MA, USA) and leukemia burden was monitored by bioluminescence imaging. The mice were anesthetized by isoflurane (RWD life science, Shenzhen, China) and imaged in the IVIS bioluminescence imager (PerkinElmer) system to assess disease burden. Analysis and normalization of in vivo luminescence was performed using Living Image v4.7 software. The investigator responsible for assessing the experimental outcomes was blinded to the group allocation. Mice were randomly assigned to treatment groups by an independent researcher who was not involved in data collection or analysis.

Six to eight weeks old *Flt3*^*ITD/ITD*^ mice were treated with vehicle, fatostatin (15 mg/kg i.p. daily), quizartinib (1 mg/kg oral daily), or quizartinib plus fatostatin for 7 days. The mice were sacrificed and bone marrow cells were collected for subsequent experiments. Cells were stained with BODIPY 493/503 and PerCP-eFluor™ 710 mouse anti-FLT3 antibody for flow cytometry analysis.

### Flow cytometric assessment of leukemia engraftment by GFP immunofluorescence

In the animal experiment of quizartinib plus fatostatin, 3 mice from each group were randomly selected and sacrificed by cervical dislocation on day 12 and day 30. The spleens dissected from mice were disrupted with a syringe plunger and filtered through a Falcon 75 μm Cell Strainer (Corning Incorporated, Corning, NY, USA) to obtain single splenocytes. Bone marrow cells were collected from femur by flushing bone cavities with RPMI 1640 and filtered by Cell Strainer. The splenocytes and bone marrow cells were resuspended in 2 mL ACK Lysing Buffer (Gibco) for 5 min to lyse red blood cells. Cell suspension was washed twice with PBS. GFP positive cells were detected by flow cytometry (Beckman Coulter, Miami, FL, USA).

### Survival analysis

Patients from TCGA database were divided into two groups according to expression of lipid genes. The surv cutpoint function of R package survminer (v.0.4.9) was used to determine the optimal cut-off gene expression value. The overall survival (OS) was analyzed by Kaplan–Meier method and log-rank test using survival (v.3.5-7) and surviminer packages.

### Data analysis

TCGA_LAML sequence data was downloaded from TCGA GDC portal (https://portal.gdc.cancer.gov/). GSE202222 and GSE163926 cohorts were obtained from Gene Expression Omnibus (https://www.ncbi.nlm.nih.gov/geo/). The gene differential expression analysis was performed using limma package (v.3.58.1) and limma voom method. The clusterProfiler (v4.10.1) package was used for KEGG enrichment and GSEA analysis.

### Statistical analysis

GraphPad Prism (v9.5.1) and R (v4.3.2) were used for statistical analysis. Shapiro–Wilk test was used to test normal distribution of the TCGA data. Welch’s test was used to calculate *p* value in the gene expression comparison between FLT3/WT and FLT3/ITD in TCGA_LAML cohort. Two-way ANOVA analysis was used to compare multiple groups. Unless otherwise specified, two-tailed unpaired Student’s *t* test was used to compare two groups. *p < 0.05. **p < 0.01. ***p < 0.001. ****p < 0.0001. The results are presented as mean values ± standard deviation (SD).

## Supplementary information

Supplementary material

Raw western blot files

## Data Availability

The data supporting the findings of this study are available from the corresponding author upon reasonable request.
