# Supplementary material for: Targeting oncogenic activation of FLT3/SREBP/FASN promotes the therapeutic effect of quizartinib involving disruption of mitochondrial phospholipids
Source: Cell Death Dis. 2025 Apr 22;16(1):327. doi: 10.1038/s41419-025-07661-6 (PMC12015539; doi:10.1038/s41419-025-07661-6)
Supplement: Supplementary file 1 — Supplementary material [file 41419_2025_7661_MOESM1_ESM.docx]

**Targeting oncogenic activation of FLT3/SREBP/FASN promotes the therapeutic effect of quizartinib involving disruption of mitochondrial phospholipids**

**Supplementary Method**

1. **Real-time quantitative PCR (qPCR)**

Total RNA was extracted using EZ-press RNA Purification Kit (EZBioscience, EZB, Roseville, MN, USA). Complementary DNA (cDNA) was synthesized using Color Reverse Transcription Kit (with gDNA Remover) (EZB). 2 x SYBR Green Pro Taq HS Premix (Accurate Biology, AG, Changsha, China) was used for target genes amplification. The LightCycler® 480 Instrument (Roche, Basel, Switzerland) was used to detect DNA amplification in real time. Sequence of the primer pairs synthesized by Sangon Biotech (Shanghai, China) are shown in Supplementary Table 2.

1. **Antibodies and reagents**

The following antibodies were used for immunoblotting or immunoprecipitation: mouse anti-SREBP1 (Santa Cruz Biotechnology, Santa Cruz, CA, USA), rabbit anti-FASN (Cell Signaling Technology, CST, Danvers, MA, USA), rabbit anti-FLT3 (CST), rabbit anti-phospho-FLT3 (Tyr589/591) (CST), rabbit anti-pan AKT (CST), rabbit anti-phospho-AKT (Ser473) (CST), mouse anti-GSK3β (Abmart, Shanghai, China), rabbit anti-phospho GSK3β (Ser9) (CST), rabbit anti-ubiquitin (CST), rabbit anti-pan phospho-Ser/Thr (Abmart), rabbit anti-β-Actin (CST), rabbit anti-α-Tubulin (CST), rabbit anti-Vinculin (CST), rabbit anti-LaminB1 (Proteintech Group, Chicago, IL, USA). The following compounds were used for in vitro or in vivo analysis: fatostatin and quizartinib (Selleck, Houston, TX77014, USA)); cycloheximide (CHX) (MedChemExpress); MG132 (Selleck); MK2206 (Selleck); CHIR-99021 (Selleck). The specific information of theses antibodies and reagents can be found in Supplementary Table 3.

1. **Measurement of cell death, mitochondrial membrane potential, mitochondrial mass and neutral lipid.**

After appropriate treatment, the number of cell death was analyzed by double staining with annexin V (4A Biotech, Suzhou, China) and Propidium iodide (BD Biosciences, Franklin Lakes, NJ, USA). Cell death percentage was detected by CytoFLEX flow cytometer (Beckman). Cells were also stained with Rhodamine 123 (0.5µM) (Sigma-Aldrich, Saint Louis, MO, USA) and mitochondrial tansmembrane potential was detected by CytoFLEX. Cells were also stained with MitoTracker^TM^ Green (0.5µM) (Thermo) and BODOPY 493/503 (1µM) for fluorescence intensity detection of mitochondrial mass and neutral lipid, respectively.

1. **Knockdown by ShRNA**

For gene knockdown experiments, leukemia cells were transfected with plko.1-shRAN-puro plasmids and selected by 1μg/mL puromycin. shRNA sequences are shown in Supplementary Table4.

**Supplementary Figures:**

**A B**

**
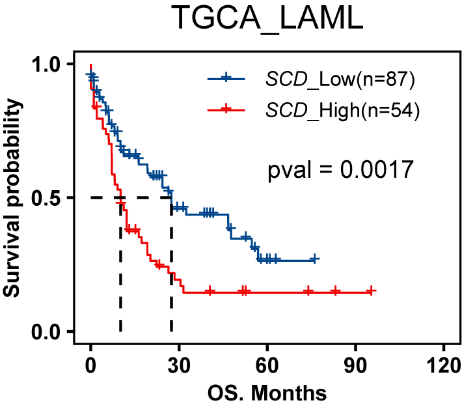

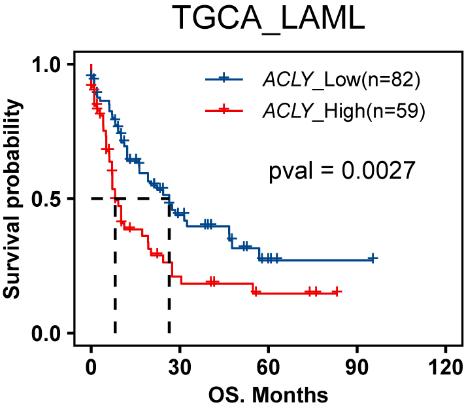
**

**Figure 1.** Kaplan-Meier analysis with log-rank test of overall survival for AML patients with low and high *ACLY* (**A**), *SCD* (**B**) expression in the TCGA_LAML cohort.

**A**

**B C**

**Figure 2.** (**A**) The association between *SREBF2* and its target genes with the overall survival of patients in the TCGA AML cohort. (**B**) Normalized log2 counts-per-million (CPM) gene-expression values of *DHCR7*, *SQLE*, and *LSS* in FLT3-WT (n=103) and FLT3-ITD (n=34) patients from the TCGA_LAML cohort. (**C**) Normalized log2 CPM gene-expression of *Dhcr7*, *Sqle*, and *Lss* in FLT3-ITD-ON vs. FLT3-ITD-OFF cells from GSE163926 dataset. Bars represent min to max value, n=3 per group.

**A B**

**C D**

**Figure 3.** (**A-B**) Comparison of SREBP1 protein expression in FLT3/ITD cells before and after treatment of MK2206 (5μM) and CHIR-99021 (3μM) for 6 and 12 hours. (**C-D**) Comparison of phosphorylated-AKT and GSK3β protein expression in in FLT3/ITD cells before and after treatment of quizartinib for 6 and 12 hours (10nM). Q, quizartinib.

**
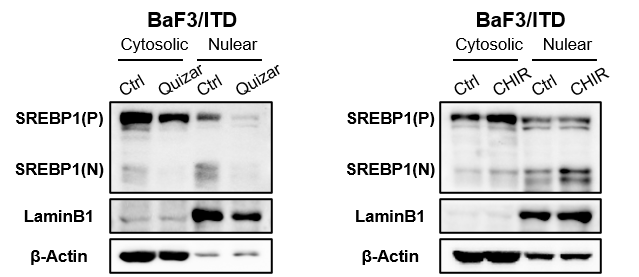
**

**Figure 4.** Subcellular localization of SREBP1 protein expression in BaF3/ITD cells before and after treatment of quizartinib (10nM) and CHIR-99021 (3μM) for 6 hours. P, precursor form. N, nuclear form. LaminB1 was used for loading control of nuclear proteins. β-Actin was used for loading control of cytosolic proteins.


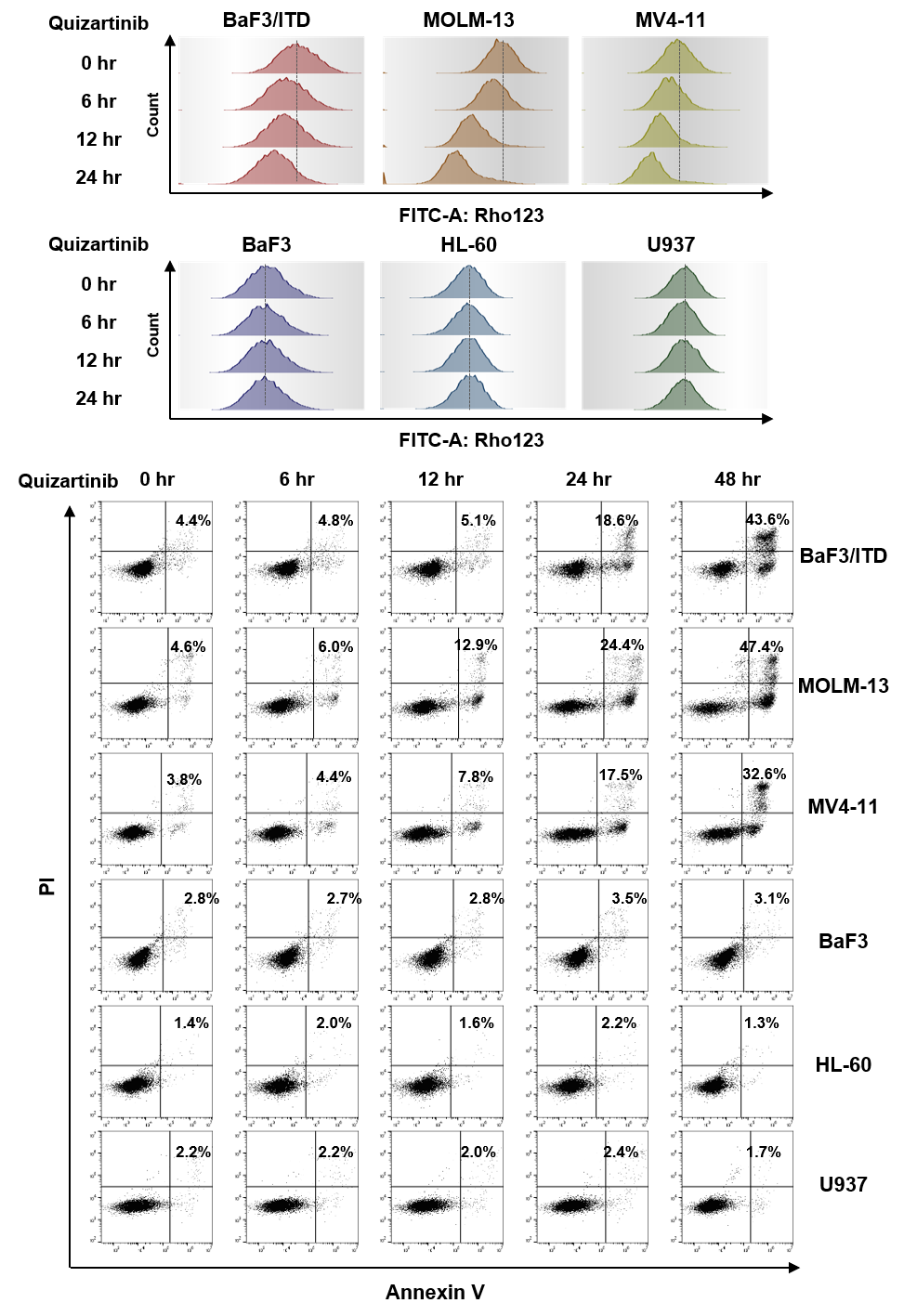
**A**

**B**

**Figure 5. (A)** Histogram of mitochondrial membrane potential of FLT3-WT (lower panel) and FLT3/ITD cells (upper panel) with treatment of quizartinib from 6-24 hours. Mitochondrial membrane potential was detected by fluorescent probe Rho123. (**B**) Cell death analysis of FLT3-WT and FLT3/ITD cells with treatment of quizartinib from 6-48 hours. Cell death was detected by annexin-V/PI.

**
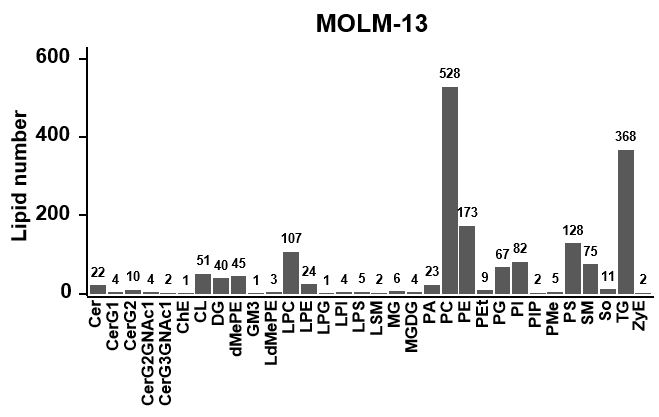
A**


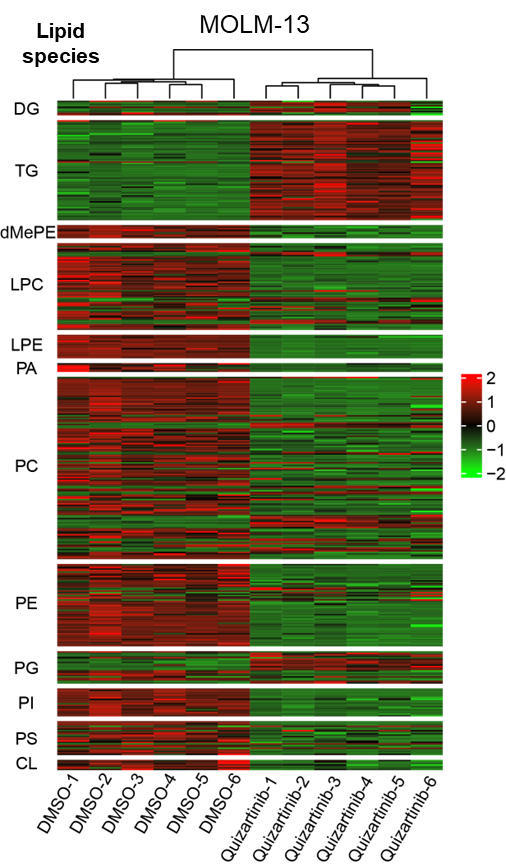


**B**

**Figure 6. (A)** Numbers of each lipid species detected by lipidomics in MOLM-13 cells. (**B**) Cluster analysis of lipidomic results in MOLM-13 cells treated with DMSO vs. quizartinib. Names of top 12 ranked differential lipid species are noted (p < 0.05, |Log2FC| > 1 and VIP > 1)

**
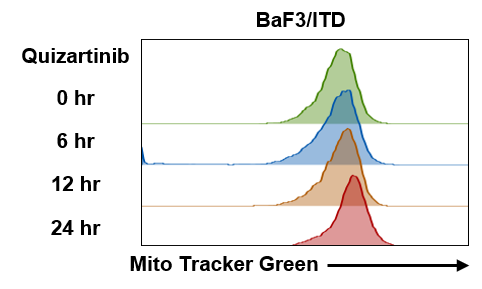
A**

**B**

**Figure 7.** (**A**) Histogram of MitoTracker Green intensity in FLT3/ITD cells before and after treatment of quizartinib from 6-24 hours. (**B**) Statistical analysis of MitoTracker Green intensity in FLT3/ITD cells before and after treatment of quizartinib from 6-24 hours. n= 3 replicates. Bars, means ± S.D. ns, non-significant.

**A**

**B**

**C**

**Figure 8.** (**A**) Annexin-V/PI assay for detection of cell death induced by different compounds in FLT-wild type (BaF3, HL-60) and FLT3/ITD (BaF3/ITD, MOLM-13) cells. Fato, fatostain (5 μM). Orlis, orlistat (10 μM). Quizar, quizartinib (10 nM). (**B-C**) Annexin-V/PI assay for detection of cell death in BaF3/ITD and MOLM-13 cells following quizartinib treatment, with or without SREBP and FASN knockdown by shRNA. Cells were exposed to the treatments for 48 hours. Numbers indicate cell death percentage of total cell number.

**
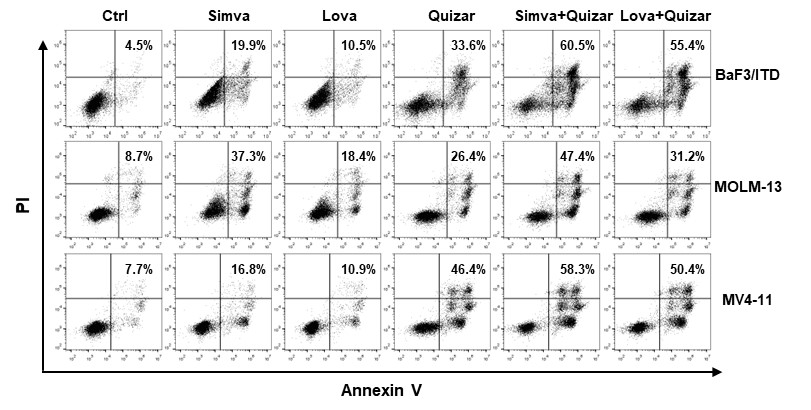
**

**Figure 9.** (**A**) Annexin-V/PI assay for detection of cell death induced by different compounds in FLT3/ITD mutated (BaF3/ITD, MOLM-13, MV4-11) cells. Simva, simvastatin (0.5 μM for BaF3/ITD and 3 μM for MOLM-13 and MV4-11). Lova, lovastatin (0.5 μM for BaF3/ITD and 3 μM for MOLM-13 and MV4-11). Quizar, quizartinib (10 nM). Cells were exposed to the treatments for 48 hours. Numbers indicate cell death percentage of total cell number.

**A**


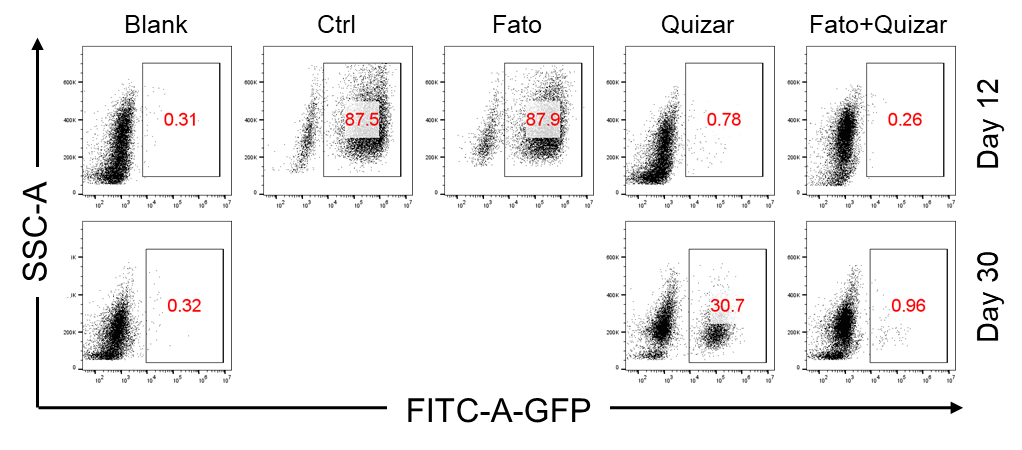


**B C**

**
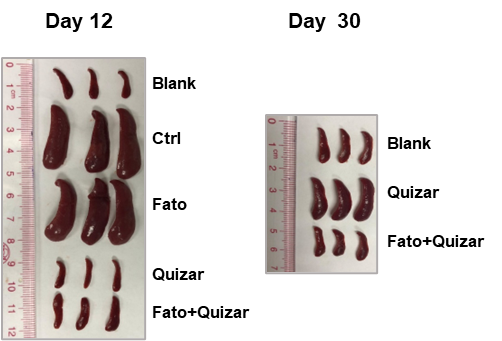
D**

**Figure 10. (A)** Infiltration of BaF3/ITD-GFP in bone marrow from mice bearing BaF3/ITD with treatment indicated on Day 12 and Day 30. Numbers indicate percentage of BaF3/ITD-GFP cells detected by flow cytometric analysis. (**B-C**) Quantitative analysis of BaF3/ITD infiltration in bone marrow and spleen on Day 12 and Day 30. (**D**) Representative images showing splenomegaly in leukemic mice on Day 12 and Day 30. Blank, normal mice without BaF3/ITD cells injection. Ctrl, control mice with BaF3/ITD leukemia. Fato, leukemic mice treated with fatostatin. Quizar, leukemic mice treated with quizartinib. Fato+Quizar, leukemic mice treated with combination of fatostatin and quizartinib.

**A**

**
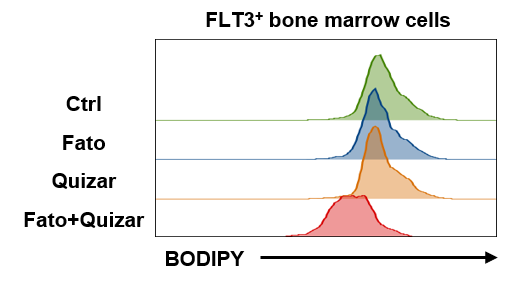
**

**B**

**Figure 11.** (**A**) Histogram of BODIPY intensity in FLT3^+^ bone marrow cells from *Flt3^ITD/ITD^* mice with different treatment. (**B**) Statistical analysis of BODIPY intensity in FLT3^+^ bone marrow cells with different treatment. N = 3 mice. Bars, means ± S.D.

**Supplementary Table 1.** Abbreviations of lipid species and enzymes in the lipid metabolism pathway.

| **Abbreviations** | **Full_name** |
| --- | --- |
| CDP-DG | CDP-diacylglycerol |
| CDS | CDP-diacylglycerol synthase |
| Cer | ceramide |
| CerG1 | monogylcosylceramide |
| CerG2 | diglycosylceramide |
| CerG2GNAc1 | dihexosyl N-acetylhexosyl ceramide |
| ChE | cholesterol ester |
| CL | cardiolipin |
| CPT | phosphocholine cytidylyltransferase |
| CRLS1 | cardiolipin synthase 1 |
| DG | diglyceride |
| dMePE | dimethyl-phosphatidylethanolamine |
| FA-CoA | fatty acids-CoA |
| G3P | glycerol-3-phosphate |
| LdMePE | lysodi-methylphosphatidylethanolamine |
| LPE | lyso-phosphatidylethanolamine |
| LPI | lyso-phosphatidylinositol |
| LSM | lyso-sphingomyelin |
| MGDG | monogalactosyldiacylglycerol |
| PA | Phosphatidic acid |
| PAP | phosphatidic acid phosphohydrolase |
| PC | phosphatidylcholine |
| PE | phosphatidylethanolamine |
| PEMT | phosphatidylethanolamine *N*-methyltransferase |
| PEt | phosphatidylethanol |
| PG | Phosphoglyceride |
| PI | phosphatidylinositol |
| PIP | phosphatidylinositol-4-phosphate |
| PIS | phosphatidylinositol synthase |
| PMe | phosphatidylmethanol |
| PS | phosphatidylserine |
| PSD | phosphatidylserine decarboxylase |
| PSS1 | phosphatidylserine Synthase-1 |
| TG | triglyceride |

**Supplementary Table 2.** Primer sequences for PCR analysis.

| **Gene ID** | **Organisms** | **primer** |  | **Sequence(5'-3'）** |
| --- | --- | --- | --- | --- |
| 60 | *Homo sapiens* | *ACTB* | Forward | CACCATTGGCAATGAGCGGTTC |
| 60 | *Homo sapiens* | *ACTB* | Reverse | AGGTCTTTGCGGATGTCCACGT |
| 6720 | *Homo sapiens* | *SREBF1* | Forward | ACAGTGACTTCCCTGGCCTAT |
| 6720 | *Homo sapiens* | *SREBF1* | Reverse | GCATGGACGGGTACATCTTCAA |
| 47 | *Homo sapiens* | *ACLY* | Forward | GCTCTGCCTATGACAGCACCAT |
| 47 | *Homo sapiens* | *ACLY* | Reverse | GTCCGATGATGGTCACTCCCTT |
| 31 | *Homo sapiens* | *ACACA* | Forward | TTCACTCCACCTTGTCAGCGGA |
| 31 | *Homo sapiens* | *ACACA* | Reverse | GTCAGAGAAGCAGCCCATCACT |
| 2194 | *Homo sapiens* | *FASN* | Forward | TTCTACGGCTCCACGCTCTTCC |
| 2194 | *Homo sapiens* | *FASN* | Reverse | GAAGAGTCTTCGTCAGCCAGGA |
| 6319 | *Homo sapiens* | *SCD* | Forward | CCTGGTTTCACTTGGAGCTGTG |
| 6319 | *Homo sapiens* | *SCD* | Reverse | TGTGGTGAAGTTGATGTGCCAGC |
| 11461 | *Mus musculus* | *Actb* | Forward | CATTGCTGACAGGATGCAGAAGG |
| 11461 | *Mus musculus* | *Actb* | Reverse | TGCTGGAAGGTGGACAGTGAGG |
| 20787 | *Mus musculus* | *Srebf1* | Forward | CCTGCCTTCAGGCTTCTCAGG |
| 20787 | *Mus musculus* | *Srebf1* | Reverse | GAGGCCAAGCTTTGGACCTGG |
| 104112 | *Mus musculus* | *Acly* | Forward | TTCGTCAAACAGCACTTCC |
| 104112 | *Mus musculus* | *Acly* | Reverse | ATTTGGCTTCTTGGAGGTG |
| 107476 | *Mus musculus* | *Acaca* | Forward | TGAAGGGCTACCTCTAATG |
| 107476 | *Mus musculus* | *Acaca* | Reverse | TCACAACCCAAGAACCAC |
| 14104 | *Mus musculus* | *Fasn* | Forward | ATTGCATCAAGCAAGTGCAG |
| 14104 | *Mus musculus* | *Fasn* | Reverse | GAGCCGTCAAACAGGAAGAG |
| 20249 | *Mus musculus* | *Scd1* | Forward | GCAAGCTCTACACCTGCCTCTT |
| 20249 | *Mus musculus* | *Scd1* | Reverse | CGTGCCTTGTAAGTTCTGTGGC |
| 14255 | *Mus musculus* | *Flt3* | Common_Forward | TCTGGTTCCATCCATCTTCC |
| 14255 | *Mus musculus* | *Flt3* | Wild type_Reverse | AGGAAGTCGATGTTGGCACT |
| 14255 | *Mus musculus* | *Flt3* | Mutant_Reverse | TGGCTACCCGTGATATTGCT |

**Supplementary Table 3.** Source of reagents and antibodies

| **Antibodies** | **Source** | **Lot** |
| --- | --- | --- |
| Rabbit anti-FLT3 | Cell Signaling Technology | #3462S |
| Rabbit anti-Phospho-FLT3 (Tyr589/591) | Cell Signaling Technology | #3464S |
| Rabbit anti-Pan AKT | Cell Signaling Technology | #4691S |
| Rabbit anti-Phospho-AKT (Ser473) | Cell Signaling Technology | #4060S |
| Mouse anti-GSK3β | Abmart | #T40069 |
| Rabbit anti-Phospho-GSK3β (Ser9) | Cell Signaling Technology | #9336S |
| Mouse anti-SREBP1 | Santa Cruz Biotechnology | #sc-13551 |
| Rabbit anti-FASN | Cell Signaling Technology | #3180S |
| Rabbit anti-Ubiquitin | Cell Signaling Technology | #3933S |
| Rabbit anti-Pan Phospho-Ser/Thr | Abmart | #T91067 |
| Mouse anti-lgG1 Isotype | Cell Signaling Technology | #5415S |
| Rabbit anti-β-Actin | Cell Signaling Technology | #8457S |
| Rabbit anti-α-Tubulin | Cell Signaling Technology | #2144S |
| Rabbit anti-α-Vinculin | Cell Signaling Technology | #13901S |
| Rabbit anti-LaminB1 | Proteintech | #12987-1-AP |
| Goat anti-rabbit IgG Antibody HRP conjugated | Signalway Antibody | #L3012 |
| Goat anti-mouse IgG Antibody HRP conjugated | Signalway Antibody | #L3032 |
| PerCP-eFluor™ 710 Mouse anti-CD135 (FLT3) Antibody | Thermo Fisher Scientific | #46-1351-82 |
|  |  |  |
| **Reagents and chemicalss** | **Source** | **Lot** |
| Quizartinib | Selleck | #S1526 |
| Fatostatin HBr | Selleck | #S8284 |
| Orlistat | Selleck | #S1629 |
| Lovastatin | Selleck | #S2061 |
| Simvastatin | MedchemExpress | #HY-17502 |
| MK-2206 2HCl | Selleck | #S1078 |
| CHIR-99021 | Selleck | #S1263 |
| Cycloheximide,CHX | MedchemExpress | #HY-12320 |
| MitoTracker™ Green FM | Thermo Fisher Scientific | #M7514 |
| BODIPY® 493/503 | Thermo Fisher Scientific | #D-3922 |
| MG-132 | Selleck | #S2619 |
| Protein A/G Magnetic Beads | MedchemExpress | #HY-K020 |
| Cardiolipin Assay Kit (Fluorometric) | BioVision | #K944 |
| NE-PER Nuclear and Cytoplasmic  Extraction Reagents | Thermo Fisher Scientific | #78835 |

**Supplementary Table 4.** Sequence of gene knockdown

| **Organisms** | **Gene**  **symbol** | **Notes** | **Sequence (5'-3')** |
| --- | --- | --- | --- |
| Homo sapiens | *GSK3β* | shRNA sequence#1 | CCGGCATGAAAGTTAGCAGAGACAACTCG AGTTGTCTCTGCTAACTTTCATGTTTTTG |
| Homo sapiens | *GSK3β* | shRNA sequence#2 | CCGGCCCAAACTACACAGAATTTAACTCG AGTTAAATTCTGTGTAGTTTGGGTTTTTG |
| Homo sapiens | *SREBF1* | shRNA sequence#1 | CCGGCCAGAAACTCAAGCAGGAGAACTCG AGTTCTCCTGCTTGAGTTTCTGGTTTTTG |
| Homo sapiens | *SREBF1* | shRNA sequence#2 | CCGGGCCATCGACTACATTCGCTTTCTCG AGAAAGCGAATGTAGTCGATGGCTTTTTG |
| Homo sapiens | *FASN* | shRNA sequence#1 | CCGGGCTACGACTACGGCCCTCATTCTCG AGAATGAGGGCCGTAGTCGTAGCTTTTTG |
| Homo sapiens | *FASN* | shRNA sequence#2 | CCGGCATGGAGCGTATCTGTGAGAACTCG AGTTCTCACAGATACGCTCCATGTTTTTG |
| Mus musculus | *Gsk3β* | shRNA sequence#1 | CCGGCCACAGAACCTCTTGTTGGATCTCG AGATCCAACAAGAGGTTCTGTGGTTTTTG |
| Mus musculus | *Gsk3β* | shRNA sequence#2 | CCGGCATGAAAGTTAGCAGAGATAACTCG AGTTATCTCTGCTAACTTTCATGTTTTTG |
| Mus musculus | *Srebf1* | shRNA sequence#1 | CCGGGCCTGCTATGAGGAGGGTATTCTCG AGAATACCCTCCTCATAGCAGGCTTTTTG |
| Mus musculus | *Srebf1* | shRNA sequence#2 | CCGGCATCGACTACATCCGCTTCTTCTCG AGAAGAAGCGGATGTAGTCGATGTTTTTG |
| Mus musculus | *Fasn* | shRNA sequence#1 | CCGGGCTGGTCGTTTCTCCATTAAACTCG AGTTTAATGGAGAAACGACCAGCTTTTTG |
| Mus musculus | *Fasn* | shRNA sequence#2 | CCGGCGTCTATACCACTGCTTACTACTCG AGTAGTAAGCAGTGGTATAGACGTTTTTG |
