# Supplementary material for: Targeting oncogenic activation of FLT3/SREBP/FASN promotes the therapeutic effect of quizartinib involving disruption of mitochondrial phospholipids
Source: Cell Death Dis. 2025 Apr 22;16(1):327. doi: 10.1038/s41419-025-07661-6 (PMC12015539; doi:10.1038/s41419-025-07661-6)

Figure1D

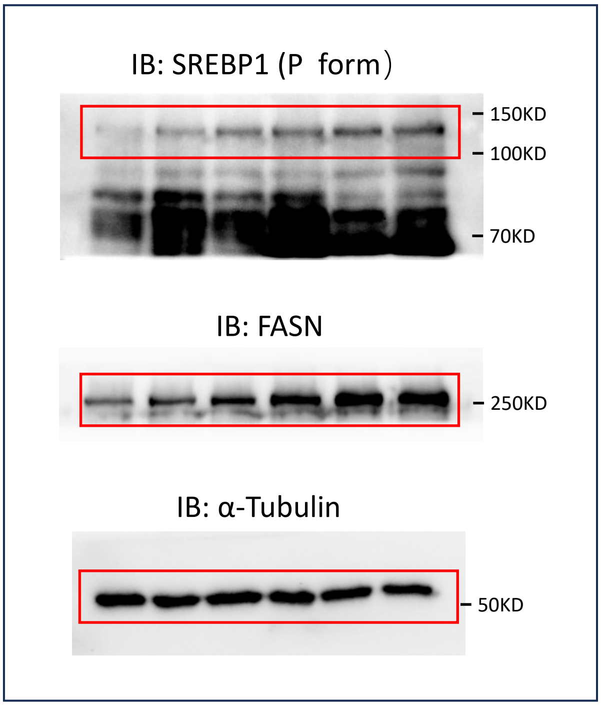

Figure1H

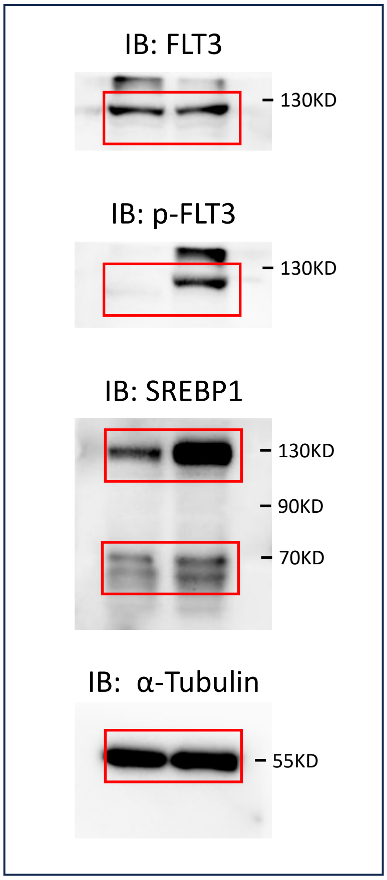

Figure3C

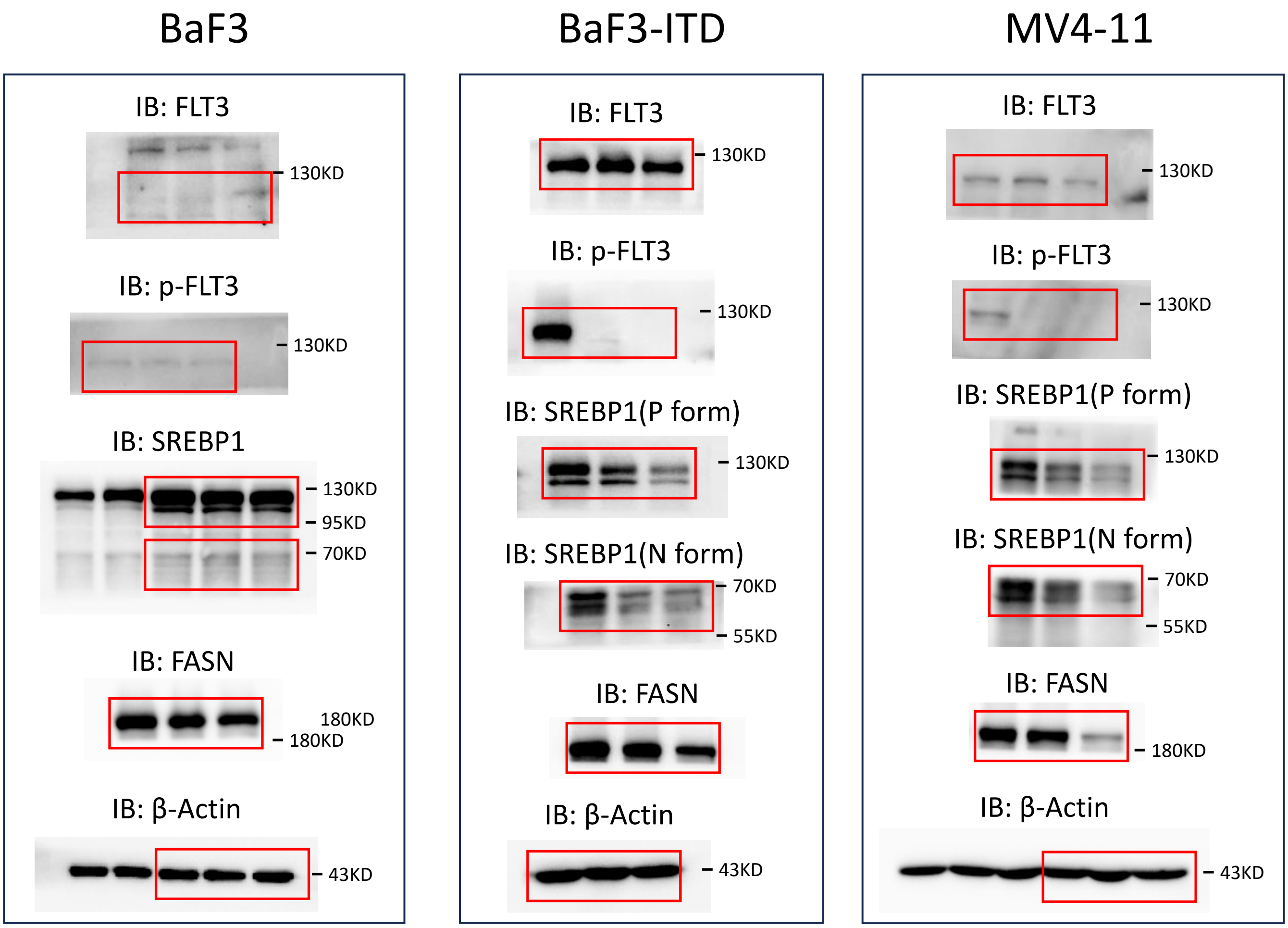
BaF3
BaF3-ITD
MV4-11
IB: FLT3
130KD
IB: p-FLT3
130KD
IB: SREBP1
130KD
95KD
70KD
IB: FASN
180KD
180KD
IB: β-Actin
43KD
IB: FLT3
130KD
IB: p-FLT3
130KD
IB: SREBP1(P form)
130KD
IB: SREBP1(N form)
70KD
55KD
IB: FASN
IB: β-Actin
43KD
IB: FLT3
130KD
IB: p-FLT3
130KD
IB: SREBP1(P form)
130KD
IB: SREBP1(N form)
70KD
55KD
IB: FASN
180KD
IB: β-Actin
43KD

Figure3C

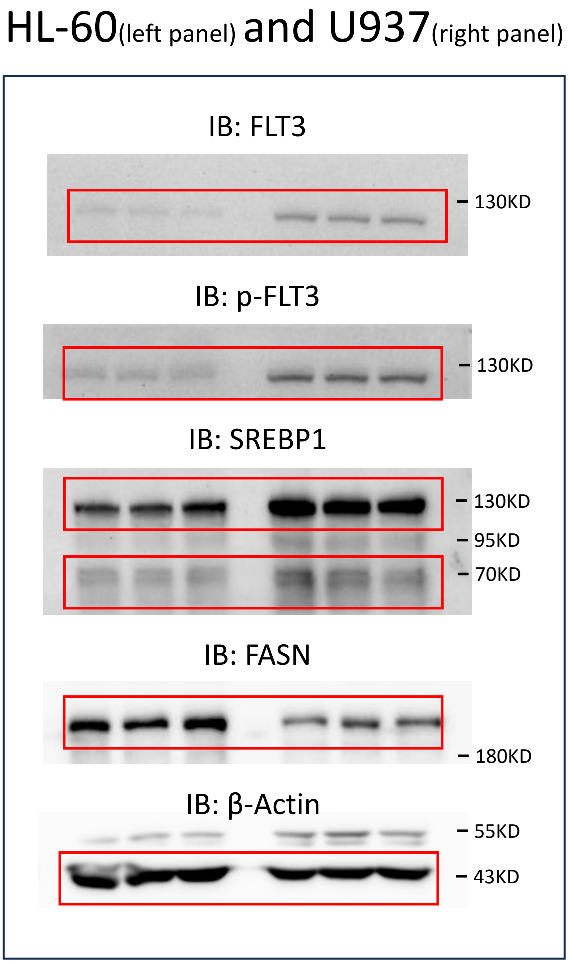

HL-60(left panel) and U937(right panel)
IB: FLT3
130KD
IB: p-FLT3
130KD
IB: SREBP1
130KD
95KD
70KD
IB: FASN
180KD
IB: β-Actin
55KD
43KD

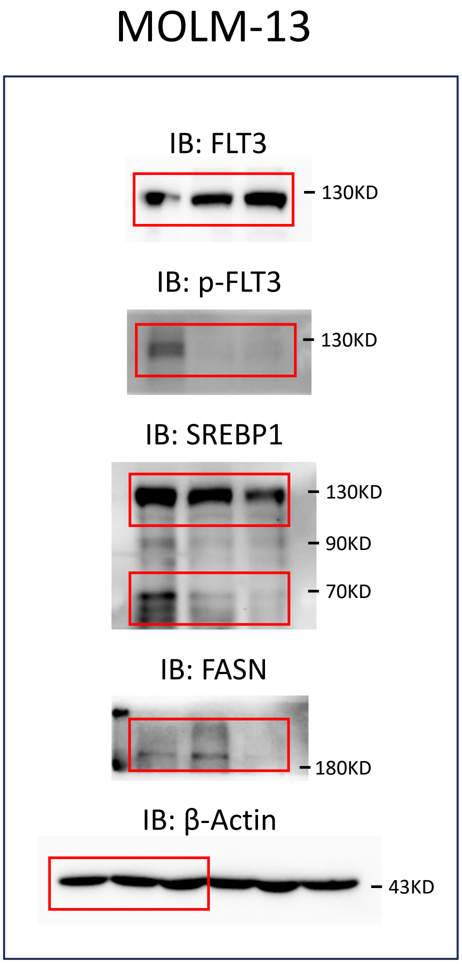

MOLM-13
IB: FLT3
130KD
IB: p-FLT3
130KD
IB: SREBP1
130KD
90KD
70KD
IB: FASN
180KD
IB: β-Actin
43KD

Figure4A

Ctrl(left panel) and Quizar_6hr(right panel)

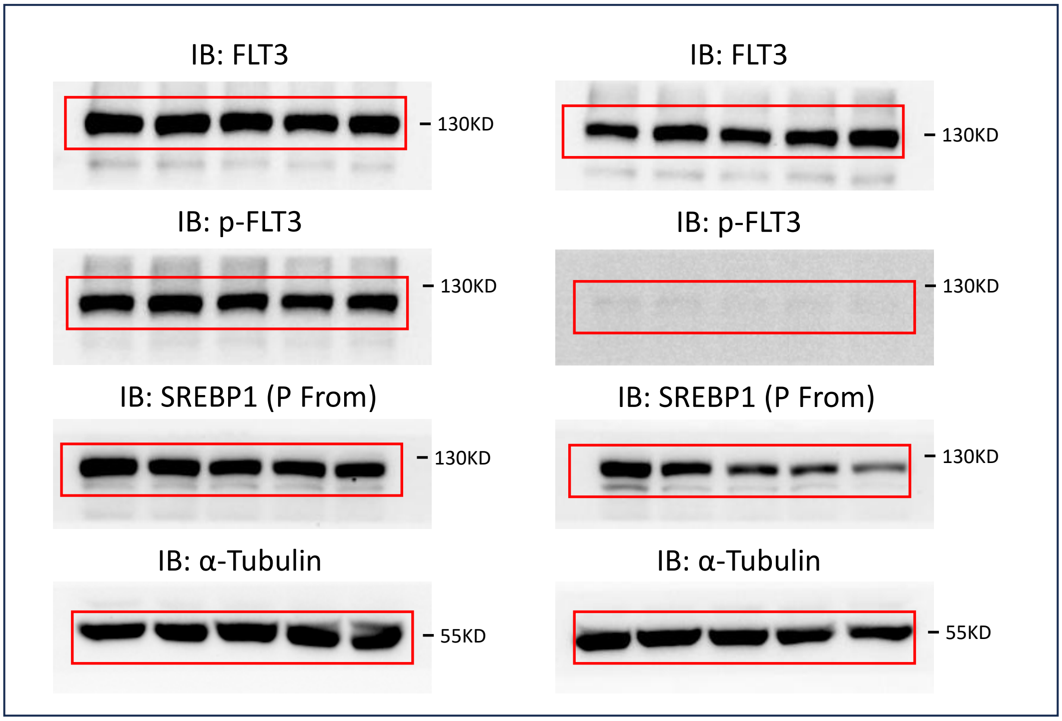

Figure4C

Ctrl(left panel) and Quizar_6hr(right panel)

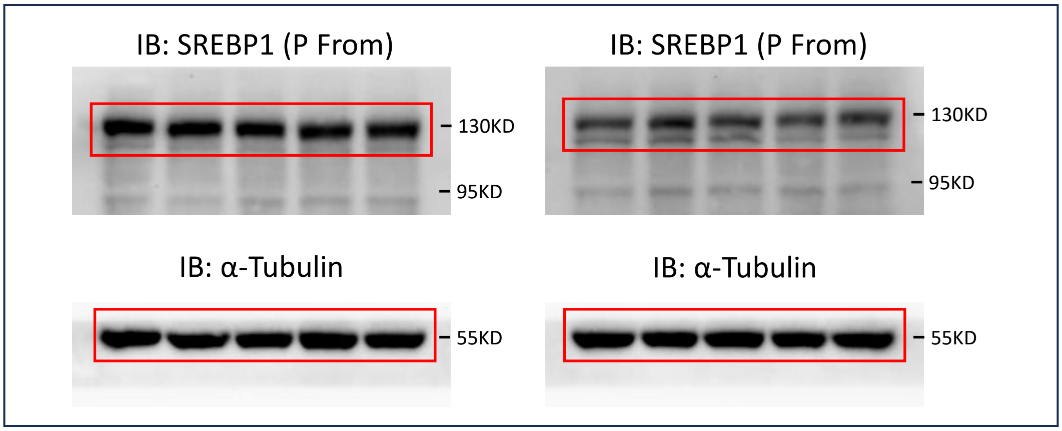

Figure4D

BaF3-ITD(left panel) and MV4-11(right panel)

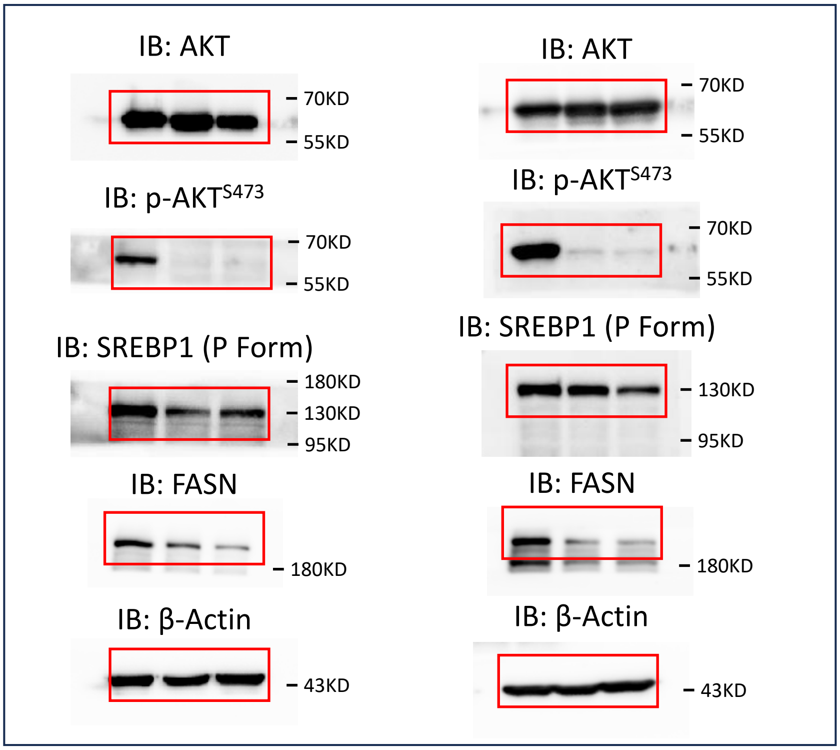

Figure4E

BaF3-ITD(left panel) and MV4-11(right panel)

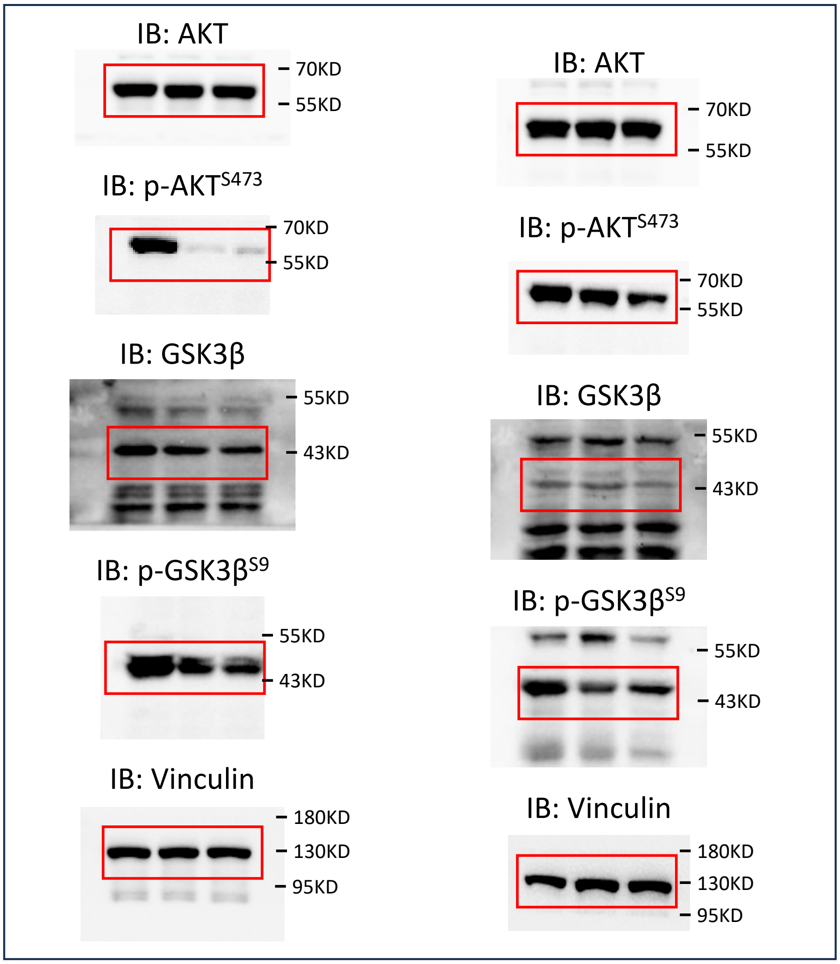

Figure4F

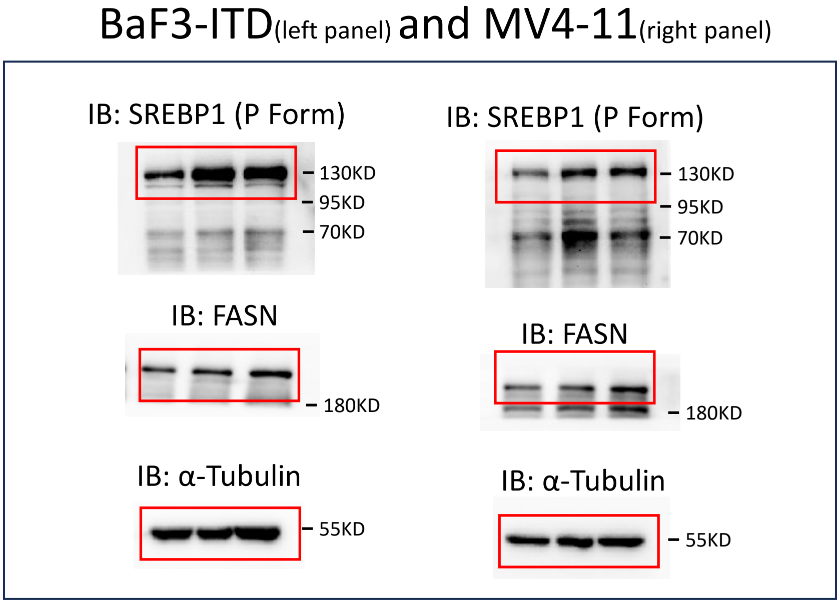

Figure4G

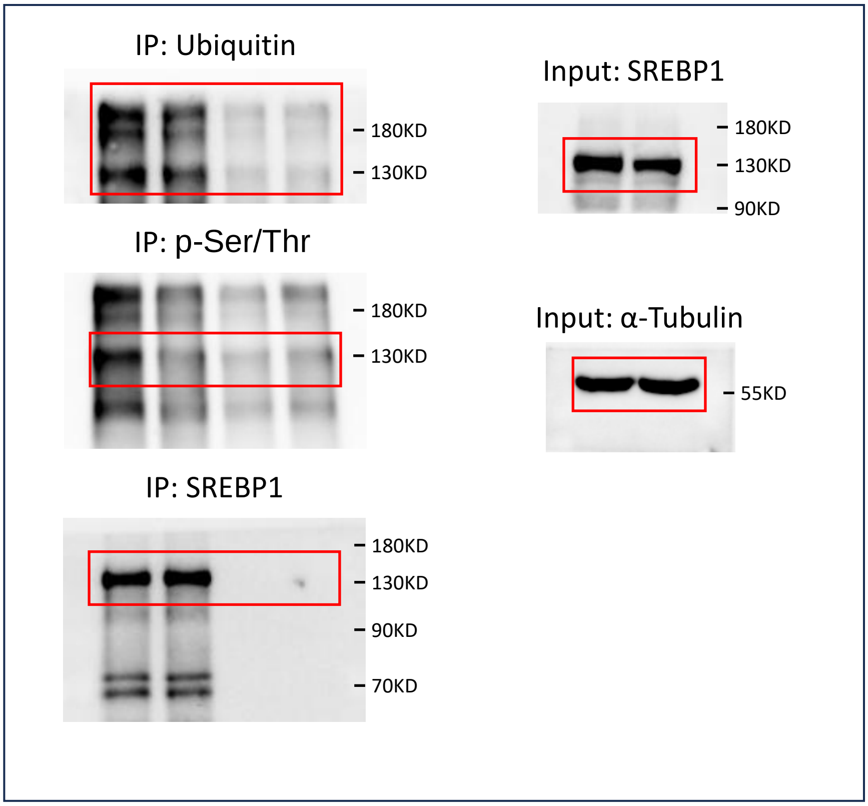

Figure4H

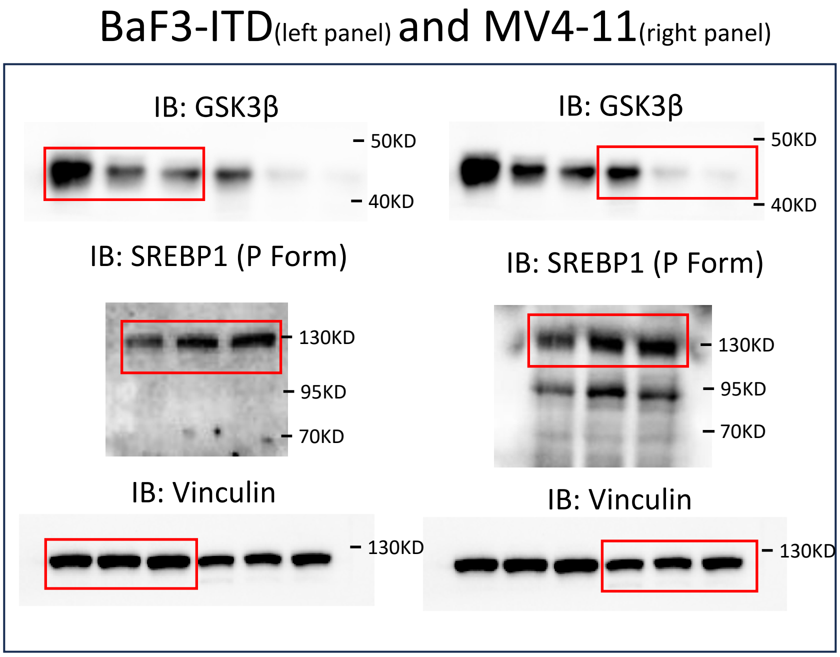

Figure4I

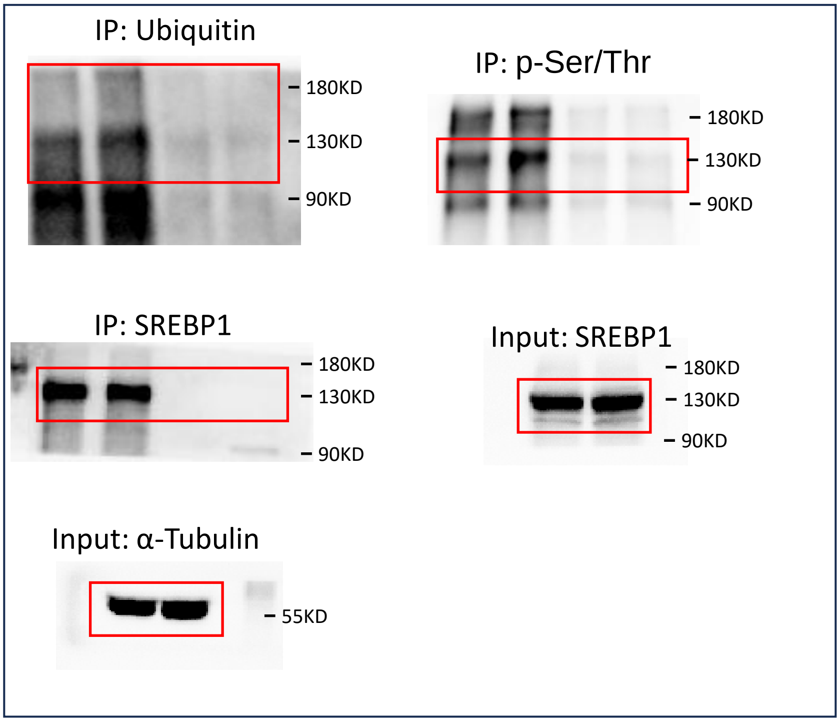

Figure6C

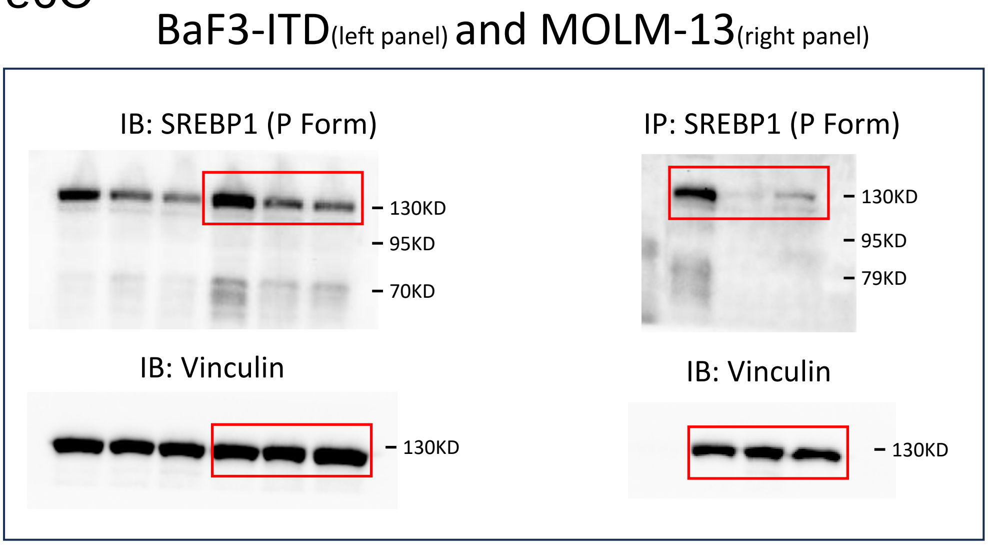

Figure6D

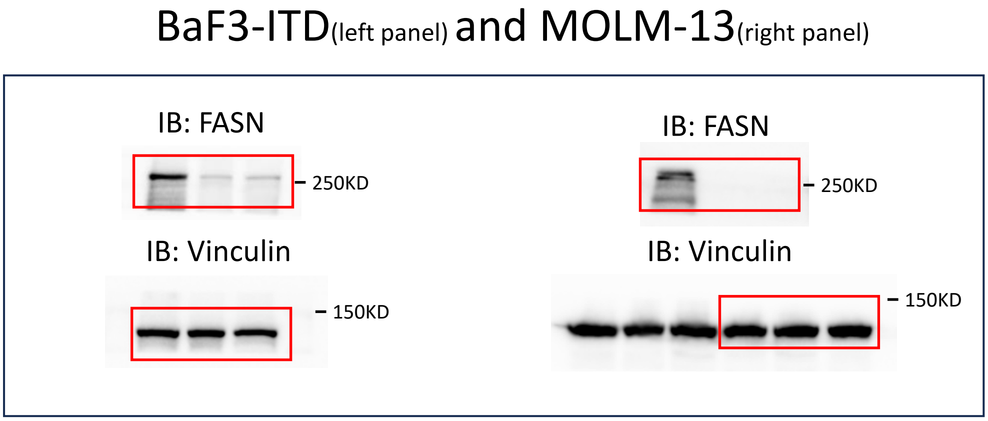

FigureS4

Quizar(left panel) and CHIR(right panel)

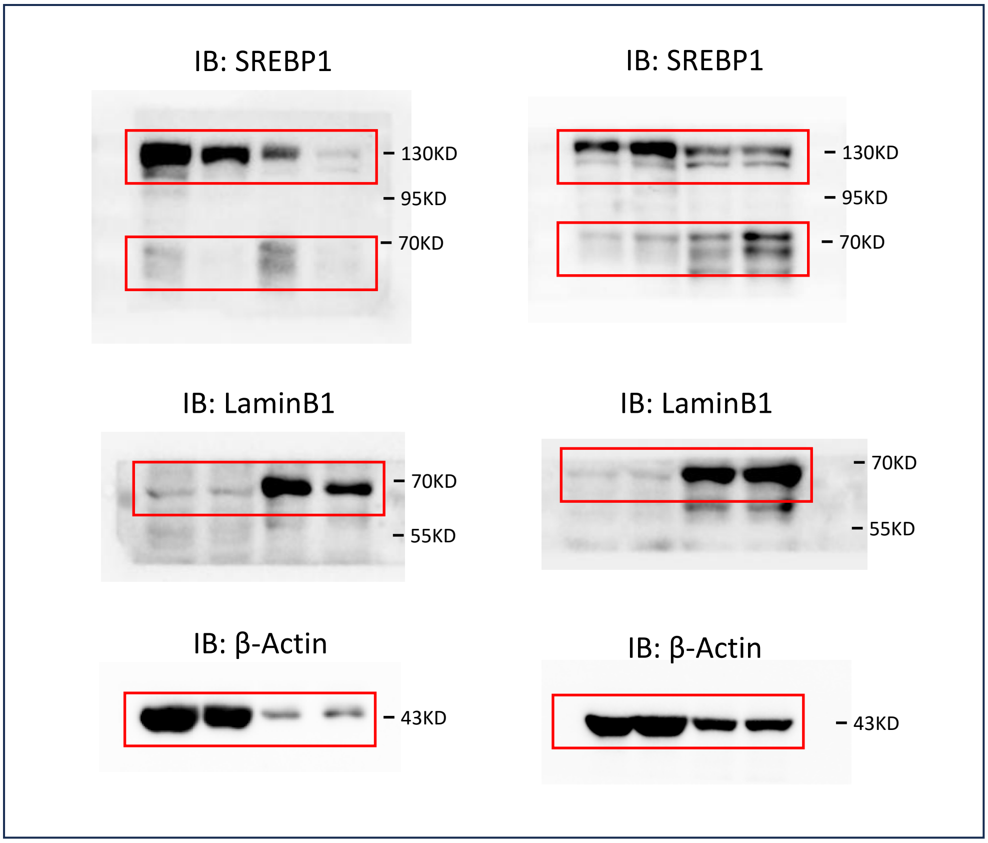

Supplement: Supplementary file 2 — Raw western blot files [file 41419_2025_7661_MOESM2_ESM.pdf]
